# Single-cell transcriptomic atlas of primate cardiopulmonary aging

**DOI:** 10.1038/s41422-020-00412-6

**Published:** 2020-09-10

**Authors:** Shuai Ma, Shuhui Sun, Jiaming Li, Yanling Fan, Jing Qu, Liang Sun, Si Wang, Yiyuan Zhang, Shanshan Yang, Zunpeng Liu, Zeming Wu, Sheng Zhang, Qiaoran Wang, Aihua Zheng, Shuguang Duo, Yang Yu, Juan Carlos Izpisua Belmonte, Piu Chan, Qi Zhou, Moshi Song, Weiqi Zhang, Guang-Hui Liu

**Affiliations:** 1 State Key Laboratory of Membrane Biology, Institute of Zoology, Chinese Academy of Sciences, Beijing, 100101 China; 2 State Key Laboratory of Stem Cell and Reproductive Biology, Institute of Zoology, Chinese Academy of Sciences, Beijing, 100101 China; 3 Institute for Stem cell and Regeneration, Chinese Academy of Sciences, Beijing, 100101 China; 4 National Laboratory of Biomacromolecules, CAS Center for Excellence in Biomacromolecules, Institute of Biophysics, Chinese Academy of Sciences, Beijing, 100101 China; 5 CAS Key Laboratory of Genomic and Precision Medicine, Beijing Institute of Genomics, Chinese Academy of Sciences, Beijing, 100101 China; 6 China National Center for Bioinformation, Beijing, 100101 China; 7 University of Chinese Academy of Sciences, Beijing, 100049 China; 8 The MOH Key Laboratory of Geriatrics, Beijing Hospital, National Center of Gerontology, Beijing, 100730 China; 9 NHC Key Laboratory of Drug Addiction Medicine, Kunming Medical University, Kunming, Yunnan 650223 China; 10 Advanced Innovation Center for Human Brain Protection, and National Clinical Research Center for Geriatric Disorders, Xuanwu Hospital Capital Medical University, Beijing, 100053 China; 11 State Key Laboratory of Integrated Management of Pest Insects and Rodents, Institute of Zoology, Chinese Academy of Sciences, Beijing, 100101 China; 12 Laboratory Animal Center, Institute of Zoology, Chinese Academy of Sciences, Beijing, 100101 China; 13 Department of Obstetrics and Gynecology, Center for Reproductive Medicine, Peking University Third Hospital, Beijing, 100191 China; 14 Stem Cell Research Center, Peking University Third Hospital, Beijing, 100191 China; 15 Gene Expression Laboratory, Salk Institute for Biological Studies, La Jolla, CA 92037 USA

**Keywords:** Senescence, Genomic analysis

## Abstract

Aging is a major risk factor for many diseases, especially in highly prevalent cardiopulmonary comorbidities and infectious diseases including Coronavirus Disease 2019 (COVID-19). Resolving cellular and molecular mechanisms associated with aging in higher mammals is therefore urgently needed. Here, we created young and old non-human primate single-nucleus/cell transcriptomic atlases of lung, heart and artery, the top tissues targeted by SARS-CoV-2. Analysis of cell type-specific aging-associated transcriptional changes revealed increased systemic inflammation and compromised virus defense as a hallmark of cardiopulmonary aging. With age, expression of the SARS-CoV-2 receptor angiotensin-converting enzyme 2 (ACE2) was increased in the pulmonary alveolar epithelial barrier, cardiomyocytes, and vascular endothelial cells. We found that interleukin 7 (IL7) accumulated in aged cardiopulmonary tissues and induced ACE2 expression in human vascular endothelial cells in an NF-κB-dependent manner. Furthermore, treatment with vitamin C blocked IL7-induced ACE2 expression. Altogether, our findings depict the first transcriptomic atlas of the aged primate cardiopulmonary system and provide vital insights into age-linked susceptibility to SARS-CoV-2, suggesting that geroprotective strategies may reduce COVID-19 severity in the elderly.

## Introduction

Advanced age renders us vulnerable to both chronic diseases and microorganismal infection.^1,2^ Common aging-related diseases such as cardiovascular disease (CVD) and chronic obstructive pulmonary disease (COPD) account for the first and third leading causes of death in the elderly population.^3,4^ Now, in a rampaging pandemic, the strong association between advanced age and a higher risk of severe illness from Coronavirus Disease 2019 (COVID-19) is of utmost medical concern.^2,5–7^ Although we still know relatively little about Severe Acute Respiratory Syndrome Coronavirus 2 (SARS-CoV-2), it has been suggested that people over the age of 65 years are more susceptible to infection^2^ and likely to die.^8^ Worldwide epidemiological studies further indicate that people with aging-related comorbidities such as COPD, CVD, and hypertension are more likely to become severely ill from COVID-19.^6,9,10^ Deepening our understanding of cellular and molecular mechanisms underlying aging may help to explain the higher vulnerability of aged individuals to SARS-CoV-2 infection, and might also identify therapeutic strategies with which we can protect the elderly from COVID-19.

Two of the most vulnerable tissues in COVID-19 are the respiratory and cardiovascular systems,^5^ in which patients experience shortness of breath and hypoxemia, lung and cardiac injury, and coagulation disorders. The lung and heart are functionally and at a cellular level very different. The primary lung function is gas exchange, but the pulmonary epithelium also acts as a physical barrier that prevents infectious agents from colonizing in the lung. The lung epithelium, technically on the outside of the body and lined with 95% of alveolar type I cells (AT1), is highly vulnerable to viral infection.^11^ The remaining fraction is made up of alveolar type II cells (AT2), which mediate the synthesis and secretion of pulmonary surfactant, with a subset of cells reported to generate new alveolar cells during lung injury.^12^ The lung also consists of capillary cells, mesenchymal cells, and a specialized set of lung-resident immune cells called alveolar macrophages (AMs) and interstitial macrophages (IMs).^13,14^ The primary function of the cardiovascular system is to maintain blood circulation to supply oxygen and nutrients throughout the body. In the heart, the cardiomyocyte (CM) is the chief cell type involved in contractile function, while endothelial and stromal cells form vessels to transport blood.^15^ Within the heart are also smooth muscle cells (SMCs), pericytes, fibroblasts, and immune cells.^15^ Given the close association between pulmonary and cardiovascular dysfunction in both aging and COVID-19, we here set out to study the aging features of these two systems in parallel. Different tissues and various cell types are heterogeneously affected by aging,^16^ suggesting the necessity of using single-cell approaches to decode the mechanisms underlying organismal aging.

Single-cell RNA sequencing (scRNA-seq) and single-nucleus RNA sequencing (snRNA-seq) have enabled the comprehensive illustration of transcriptional changes at the single-cell resolution and have been particularly powerful for revealing the unknown dynamic cellular complexity and transcriptional heterogeneity during aging.^17–20^ snRNA-seq possesses unique advantages, such as less biased cellular coverage, reduced dissociation-induced transcriptional artifacts, and applicability to frozen specimens.^17,21^ snRNA-seq is also suitable for analysis of fibrotic tissues and postnatal samples where cell isolation is difficult, like aged lung and heart under study here.^17,21^ Another advantage of scRNA-seq/snRNA-seq is that it promises the discovery of rare cell populations, such as the relatively rare subpopulation of cells that express angiotensin-converting enzyme 2 (ACE2), recently identified as a SARS-CoV-2 receptor for cell entry.^17,22,23^

ACE2 has emerged as a major determinant for SARS-CoV-2 entry and transmissibility.^22–25^ Human recombinant soluble ACE2 has been found to effectively block early-stage SARS-CoV-2 cell entry.^26^ Accessory proteases including TMPRSS2, FURIN, CTSL, and CTSB of cathepsin family members, and perhaps other lung proteases, may trigger fusion of viral and cellular membranes to facilitate the cell entry of SARS-CoV-2.^23,27–30^ Accessory proteases are expressed with relatively higher abundance and are to some extent likely interchangeable.^23^ Thus, a detailed characterization of the aging-related changes in the expression pattern and regulatory mechanism of ACE2 will help understand the patterns of transmission and spread, tissue tropism, and higher pathogenicity of SARS-CoV-2 in the elderly.

Non-human primate (NHP) has significant genetic homology with humans and exhibits similar sensitivity to aging-related human diseases, including COPD and CVD.^31,32^ Likewise, aged monkeys suffer from more severe COVID-19-like symptoms after SARS-CoV-2 infection compared to young monkeys.^33,34^ Therefore, NHP makes an ideal model for studying the fundamental mechanisms of aging and aging-associated susceptibility to viral infection. Yet, lack of a comprehensive understanding of the aging mechanisms of primate lung and cardiovascular tissues impedes the search for any potential clues that can explain the age-related severity of COVID-19.

Here, we generated the first single-nucleus transcriptomic atlas of primate lung and cardiovascular systems at young vs old ages. Our dataset revealed pathways related to primary functions that are undermined in aging, along with compromised cell-intrinsic host defense mechanisms. The senescence-associated secretory phenotype (SASP) landscape illustrated that SASP is commonly upregulated in various cell types, and pinpoints interleukin 7 (IL7) as an aging marker elevated in the aged lung, heart, and vessel. We found that the treatment of human arterial endothelial cells (HAECs) with IL7 upregulated the expression of ACE2 in an NF-κB-dependent manner, and that ACE2 was upregulated in a tissue- and cell type-specific manner in aged monkeys, particularly in arterial endothelial cells. Moreover, supplementation of vitamin C, a geroprotector, abolished the stimulation of ACE2 expression upon IL7 treatment in HAECs. Our study provides a valuable resource for studying the underlying mechanisms of aging and aging-related increased vulnerability to chronic diseases and viral infection, suggesting that geroprotective strategies may be applied to protect the elderly from greater COVID-19 severity.

## Results

### Characterization and single-nucleus transcriptome profiling of young and old primate lung and heart

**Fig. 1:**
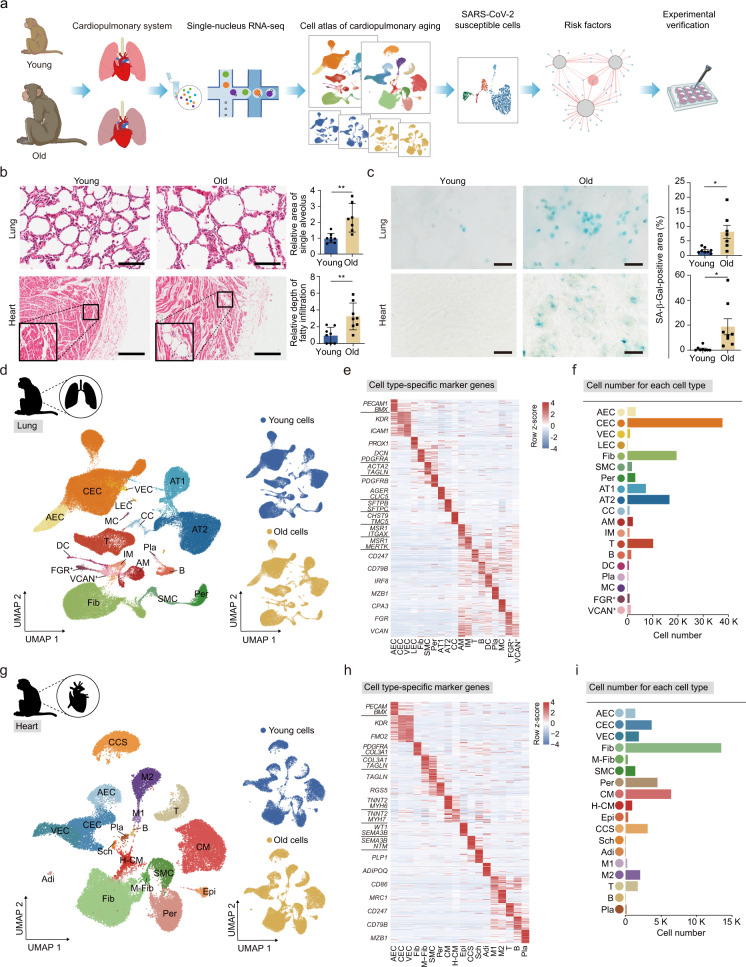
Generation of cynomolgus monkey single-nucleus RNA-seq lung and heart cell atlases. **a** Study flowchart. **b** Hematoxylin and eosin (H&E)-stained sections of lung and heart tissues from young and old monkeys. Representative images are shown on the left; quantitative data for the relative area of the single alveolus (lung) and relative depth of fatty infiltration (heart) are shown as means ± SEM on the right. Scale bar, 100 μm (lung) and 200 μm (heart). Young, *n* = 8; old, *n* = 7 monkeys (lung). Young, *n* = 8; old, *n* = 8 monkeys (heart). **P < 0.01. **c** SA-β-Gal staining of lung and heart tissues. Representative images are shown on the left; quantitative data for each tissue are shown as means ± SEM on the right. Young, *n* = 8; old, *n* = 7 monkeys (lung). Young, *n* = 8; old, *n* = 8 monkeys (heart). Scale bar, 50 μm. *P < 0.05. **d** UMAP plots showing different cell types (left) and young and old cell distribution (right) in monkey lung. AEC, arterial endothelial cell; CEC, capillary endothelial cell; VEC, venous endothelial cell; LEC, lymphatic endothelial cell; Fib, fibroblast; SMC, smooth muscle cell; Per, pericyte; AT1, alveolar type I cell; AT2, alveolar type II cell; CC, ciliated cell; AM, alveolar macrophage; IM, interstitial macrophage; T, T cell; B, B cell; DC, dendritic cell; Pla, plasmocyte; MC, mast cell; FGR^+^, *FGR*-positive cell; VCAN^+^, *VCAN*-positive cell. **e** Heatmap showing the gene expression signatures of the top 30 marker genes corresponding to each cell type in monkey lung. Each column represents one cell type, and each row indicates the expression of one gene. Marker genes for each cell type are shown on the left of the heatmap. **f** Bar plot showing the cell numbers of different cell types in monkey lung. **g** UMAP plots showing different cell types (left) and young and old cell distribution (right) in monkey heart. M-Fib, myofibroblast; CM, cardiomyocyte; H-CM, hypertrophic cardiomyocyte; Epi, epicardial cell; CCS, cardiac conduction system cell; Sch, Schwann cell; Adi, adipose cell; M1, pro-inflammatory macrophage; M2, anti-inflammatory macrophage. **h** Heatmap showing the gene expression signatures of each cell type in monkey heart. Each column represents one cell type, and each row indicates the expression of one gene. Marker genes for each cell type are shown on the left of the heatmap. **i** Bar plot showing the cell numbers of different cell types in monkey heart.

In this study, we asked how aging changes cardiopulmonary tissues at the cellular and molecular levels. To this end, we collected lung and heart tissues from young (4–6 years old) and aged (18–21 years old) cynomolgus monkeys, equivalent to young adults (~20 years old) and old humans (~70 years old), respectively (Fig. 1a; Supplementary information, Table S1). Aged monkey lung was characterized by alveolar enlargement, increased fibrosis, and fat deposition relative to young monkey lung (Fig. 1b; Supplementary information, Fig. S1a, b). Similarly, we observed fatty infiltration of the myocardium in the aged heart (Fig. 1b; Supplementary information, Fig. S1b). In addition, aged lung and heart exhibited a higher level of senescence-associated β-galactosidase (SA-β-Gal) staining, which is often featured by senescent cells (Fig. 1c).

We then performed snRNA-seq analysis of lungs and hearts from young and aged cynomolgus monkeys (Fig. 1a; Supplementary information, Table S1). Nuclei isolated from the lung and the heart of each monkey were individually loaded into a droplet-forming microfluidic device and sequenced to reach a sequencing saturation over 70% per sample (Supplementary information, Table S2). After stringent quality control,^35^ including doublet removal, a total of ~150,000 nuclei from lung and heart were obtained for downstream analysis (Supplementary information, Table S2).

Using unbiased clustering and Uniform Manifold Approximation and Projection (UMAP) analysis, we identified 19 cell types in the lung and 18 cell types in the heart with distinct cellular transcriptomic signatures (Fig. 1d–i; Supplementary information, Fig. S2a, c). To map these to known cell types, we compared gene expression profiles with classic cell type-specific markers in the heatmaps and dot plots (Fig. 1e, h; Supplementary information, Fig. S2b, d and Table S2). In the lung, we annotated AT1 and AT2, ciliated cells (CCs), four types of endothelial cells (arterial endothelial cells (AECs), capillary endothelial cells (CECs), venous endothelial cells (VECs), and lymphatic endothelial cells (LECs)), pericytes (Per), SMCs, and fibroblasts (Fib). In addition, we identified seven types of tissue-resident immune cells including AMs, IMs, B cells (B), T cells (T), mast cells (MCs), dendritic cells (DCs) and plasmocytes (Pla), and two types of monocyte-like cells (FGR^+^ and VCAN^+^ cells) (Fig. 1d). In the heart, we annotated CMs, hypertrophic cardiomyocytes (H-CMs), epicardial cells (Epi), adipose cells (Adi), two cell types related to nerve conduction (cardiac conduction system cells (CCSs) and Schwann cells (Sch)), three types of endothelial cells (AECs, CECs and VECs), four mesenchymal cell types (Fib, myofibroblasts (M-Fib), SMCs and Per). In addition, we identified five types of tissue-resident immune cells in the heart, pro-inflammatory macrophages (M1), anti-inflammatory macrophages (M2), B, T and Pla (Fig. 1g). Detailed information about marker genes for each cell type is listed in Supplementary information, Table S2.

Gene Ontology (GO) analysis of differentially expressed genes (DEGs) across cell types revealed features corresponding to known biological functions and characteristics of each cell cluster (Supplementary information, Fig. S2e, f). For instance, the GO terms “epithelial cell migration” and “regulation of cell adhesion” were specific to AT1, whereas “surfactant metabolism” and “stem cell proliferation” were specific to AT2 (Supplementary information, Fig. S2e). In the heart, GO terms “cardiac muscle tissue development” corresponded to the molecular features of CMs and “establishment of the endothelial barrier” to those of endothelial cells (Supplementary information, Fig. S2f). In addition, global transcriptomic profiling showed that cell type identity was not noticeably affected during primate aging (Fig. 1d, g; Supplementary information, Fig. S2b, d). Collectively, our data represent the first single-nucleus transcriptomic map for young versus old primate lung and heart.

### Age-related gene expression changes in different cell types of the primate lung

**Fig. 2:**
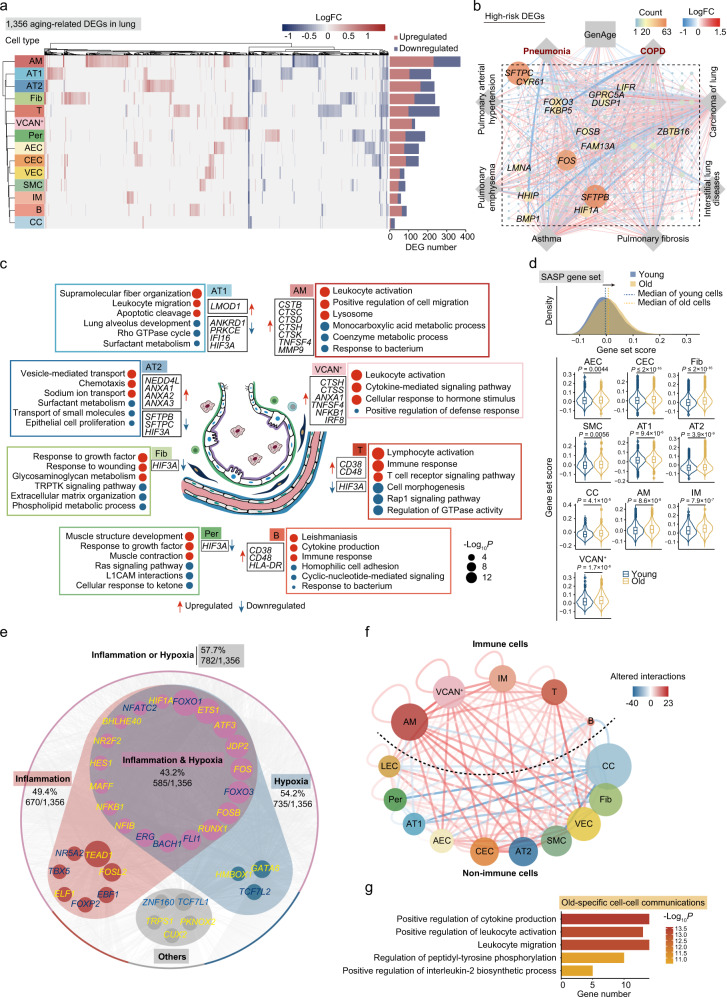
Age-related transcriptional alterations in various cell types of monkey lung. **a** Left, heatmap showing the DEGs (|logFC| > 0.25, adjusted *P* value < 0.05) during aging across different cell types in monkey lung. Right, bar plot showing the numbers of DEGs across different cell types in monkey lung. **b** Network plot showing the DEGs overlapped with GenAge database (https://genomics.senescence.info/genes/) and lung disease database (https://www.malacards.org/, https://www.disgenet.org/home/). The node size (count) indicates the number of cell types across databases in which the genes were differentially expressed with age. The color of connecting lines corresponds to the logFC. Genes with count > 20 are shown. **c** Diagram showing the enrichment of GO terms (Biological Process) or pathways in four parenchymal cell types (AT1, AT2, Fib, Per) and four immune cell types (AM, VCAN^+^, T, B) with the highest numbers of DEGs (|logFC| > 0.25, adjusted *P* value < 0.05). The dot size is positively correlated with the –log_10_ *P*-value. Representative DEGs are also shown beside the terms. Red, upregulation; Blue, downregulation. **d** Top, ridge plot showing the shift of SASP gene set score with age in all cells from monkey lung. Bottom, violin plots showing the cell types in which the SASP gene set score is significantly increased with age in monkey lung. **e** Network plot showing the upregulated and downregulated TFs during aging (|logFC| > 0.25, adjusted *P* value < 0.05) associated with inflammation, hypoxia, or other biological processes. Upregulated TFs are highlighted in yellow, and downregulated TFs are highlighted in blue. The TFs are separated into 4 groups: “inflammation” (circled by red background), “hypoxia” (circled by blue background), “inflammation & hypoxia” (circled by both red and blue background) and “others” (circled by gray background). The outer nodes represent the target genes of TFs from the group of “inflammation” (red and purple), “hypoxia” (blue and purple), “inflammation & hypoxia” (purple) or “others” (gray). The percentages represent the ratios of target DEGs to total DEGs. **f** Network plot showing the cell–cell communications between immune cells and non-immune cells in monkey lung. The color of connecting lines indicates the number of altered interaction pairs. Red, increased interactions; blue, decreased interactions. **g** Bar plot showing the enrichment of GO terms or pathways of old-specific cell–cell communications.

To elucidate molecular mechanisms associated with aging at a cellular level, we sought to identify aging-associated transcriptional changes in individual lung cells. We found that a total of 1356 DEGs (|logFoldChange(logFC)| > 0.25, adjusted *P* value < 0.05) were differentially expressed in at least one type of aged lung cells compared to their younger counterparts (Fig. 2a). The cell types most affected during aging included AMs, T cells, fibroblasts, AT2 and AT1, with 372, 262, 239, 235, and 217 DEGs in old vs young lung subtypes, respectively (Fig. 2a; Supplementary information, Fig. S3a and Table S3).

This set of identified DEGs was firstly evaluated in databases comprising hotspot genes known to be involved in aging and various lung diseases (COPD, pneumonia, pulmonary fibrosis, and asthma, etc.). An overlap of the DEGs with genes in the database indicated that aging is a major contributing factor for chronic respiratory diseases and infection (Fig. 2b; Supplementary information, Fig. S3a). GO term annotations shared across cell types revealed that downregulated DEGs are involved in epithelial cell proliferation, hemostasis, and surfactant metabolism (Fig. 2c; Supplementary information, Fig. S3b), indicative of compromised lung function during aging. Genes related to leukocyte activation and cellular response to lipids were activated in AMs, T, and B cells (Fig. 2c; Supplementary information, Fig. S3b). Additionally, SASP-related genes were found to be augmented in 10 cell types, including CECs, fibroblasts, AT1, AT2, and AMs (Fig. 2d), demonstrating that the aged lung is characterized by cellular stress and chronic inflammation. Specifically, upregulated DEGs were associated with vesicle-mediated transport (Fig. 2c; Supplementary information, Fig. S3b), which may constitute a contributor to viral internalization.

Next, we performed Single-Cell Regulatory Network Inference and Clustering (SCENIC) analysis and discovered that dozens of nodal transcription factors (TFs) were integrated into the regulatory network of aging-associated DEGs. More than half of the nodal TFs were hypoxia-responsive TFs that were activated in a cell type-specific manner during lung aging (Fig. 2e; Supplementary information, Fig. S3a and Table S4). *HIF1A*, the master regulator of hypoxia response, appeared upstream of age-related transcriptional dynamics in several cell types in the aged lung, including AMs, B cells, and AECs (Supplementary information, Fig. S3a). We also observed that another hypoxia-induced protein, HIF3A, reported to be downregulated in inflammatory states and inversely correlated with *HIF1A*,^36^ was decreased in AT1, AT2, CECs, AECs, pericytes, fibroblasts, and T cells (Supplementary information, Fig. S3c). In addition, hypoxia-inducible *ATF3*, known to be increased in idiopathic pulmonary fibrosis,^37^ appeared as a critical regulator upregulated in AT1, AT2, fibroblasts and AMs (Fig. 2e; Supplementary information, Fig. S3a). Other hypoxia-induced regulators, such as *RUNX1* (AT2 and T cells), *MAFF* (Fib), and *FOS* (AT1, AECs, B cells, etc.) were also upregulated (Fig. 2e; Supplementary information, Fig. S3a). In all, these TFs regulate more than half of age-related DEGs in the lung (Fig. 2e). At the protein level, we confirmed that the master hypoxia-inducible TF HIF1α was upregulated in the aged lung by immunofluorescence analysis, indicating that the aged lung may undergo marked hypoxia stress that probably contributes to profound aging phenotypes (Supplementary information, Fig. S3d). In line with the notion that inflammatory pulmonary phenotypes are often accompanied with hypoxic lung disease, we found that *NFKB1*, a hypoxia-induced pro-inflammatory TF,^38^ was upregulated in aged lung VECs and immune cells (Fig. 2e; Supplementary information, Fig. S3a). Altogether, our data point to a possibility that age-related impairment in alveolar structure may function as a cellular basis for alveolar hypoxia triggering an inflammatory cascade.

### Changes of local alveolar microenvironments in the aged lung

We next surveyed the transcriptional signatures of epithelial and immune cell types and predicted their cell–cell interaction changes during aging. In AT1, which make up 95% of the alveolar epithelial barrier, a panel of cytoskeletal genes related to supramolecular fiber organization was upregulated during aging (Fig. 2c). Among all upregulated DEGs of AT1, *LMOD1* (logFC = 1.09), a reported marker gene for idiopathic pulmonary fibrosis,^39^ ranked here as the most upregulated gene (Fig. 2c; Supplementary information, Fig. S3c). By comparison, the two most downregulated genes in aged AT1 were *ANKRD1* (logFC = –0.87) and *PRKCE* (logFC = –0.80), both serving as downstream factors of the TLR3/TLR4-specific antiviral gene program (Fig. 2c; Supplementary information, Fig. S3c).^40,41^ Notably, the silencing of *ANKRD1* increased viral load in antigen-presenting cells.^41^ *IFI16* (logFC = –0.26) (Fig. 2c; Supplementary information, Fig. S3c), encoding a viral DNA sensor that represses viral gene expression and replication, was also downregulated in aged AT1.^42^ In AT2, the progenitors that give rise to AT1 cells and that occupy the remaining 5% of the alveolar surface, surfactant protein-coding genes *SFTPB* (logFC = –0.45) and *SFTPC* (logFC = –0.33) were downregulated during aging (Fig. 2c; Supplementary information, Fig. S3c). By maintaining the lower surface tension, these proteins are critical to prevent lung collapse (atelectasis) during breathing. It should be particularly noted that *SFTPB* and *SFTPC*, two of the most robust susceptibility factors for lung diseases (Fig. 2b), are also critical for enhanced mucosal immunity and strong antiviral response to influenza.^43^ Additionally, we observed that 21 vesicle-mediated transport-related genes were upregulated in aged AT2, including viral budding factor *NEDD4L*^44^ and *ANXA1*, *ANXA2*, and *ANXA3* (Fig. 2c; Supplementary information, Fig. S3c), related to viral entry, assembly, and production. Therefore, these results point to a hypothesis that aging may lead to functional decay of the alveolar epithelium barrier as well as the compromised ability of viral defense.

Globally, we observed increased proportions of many immune cell types residing in the aged lung tissues (Supplementary information, Fig. S4a–d), including mast cells and plasmocytes (Supplementary information, Fig. S4a, b) that secrete cytokines and antibodies, respectively, likely contributing to increased age-related inflammation as well as COPD and idiopathic pulmonary fibrosis.^45^ In addition, CD8^*+*^ T cells that produce inflammatory cytokines and cytotoxic molecules in the pathogenesis of COPD were also elevated in the aged lung (Supplementary information, Fig. S4c). AMs function as the first line of defense against pathogens in the lung. We identified 31 upregulated genes involved in leukocyte degranulation and activation during AM aging, such as *CSTB*, *CTSC*, *CTSD*, *CTSH*, and *CTSK*. Notably, the upregulation of *TNFSF4* and *MMP9* (Fig. 2c; Supplementary information, Fig. S3c) are frequently observed in classical AM activation during an inflammatory response, indicative of a hyperactive frontier macrophage state. As for monocyte-like cells (VCAN^+^), more than 30 genes involved in leukocyte activation, such as *CTSH*, *ANXA1*, *TNFSF4*, *NFKB1*, and *IRF8*, were increased in aged monocyte-like cells (VCAN^+^), indicating low-grade chronic inflammation (Fig. 2c; Supplementary information, Fig. S3c). Similarly, we observed a panel of upregulated genes related to an abnormal immune response in B cells and T cells, including *CD38*, *CD48*, and *HLA-DR* (Fig. 2c; Supplementary information, Fig. S3c). Similar changes were reported in a 50-year-old man who succumbed to SARS-CoV-2,^46^ implying an infection-susceptible immunological lung microenvironment that facilitates initiation or amplification of virus-caused detrimental effects during aging.

Consistent with increased lymphocyte activation, the predicted frequency of global pro-inflammatory cell–cell interactions was increased in the aged lung (Fig. 2f, g; Supplementary information, Fig. S4e and Table S5), approximately half of which were related to cytokine production and leukocyte activation (e.g., TNFSF10, TNFSF13, IL1A, IL15RA, as well as TGF-β1 and TGF-β2, master regulators of lung fibrosis) (Fig. 2g; Supplementary information, Table S5). For instance, the interaction of IL1 receptor with its ligand IL1A was especially strengthened between AT2 and AM, and that the interaction of TGF-β receptor type 2 with its ligand TGF-β2 was elevated between AT1, AT2 and AM (Supplementary information, Fig. S4f and Table S5). Thus, the single-cell transcriptomics landscape reveals how inflammation reshapes the local alveolar niche during pulmonary aging.

### Age-related gene expression changes in different cell types of the primate heart

**Fig. 3:**
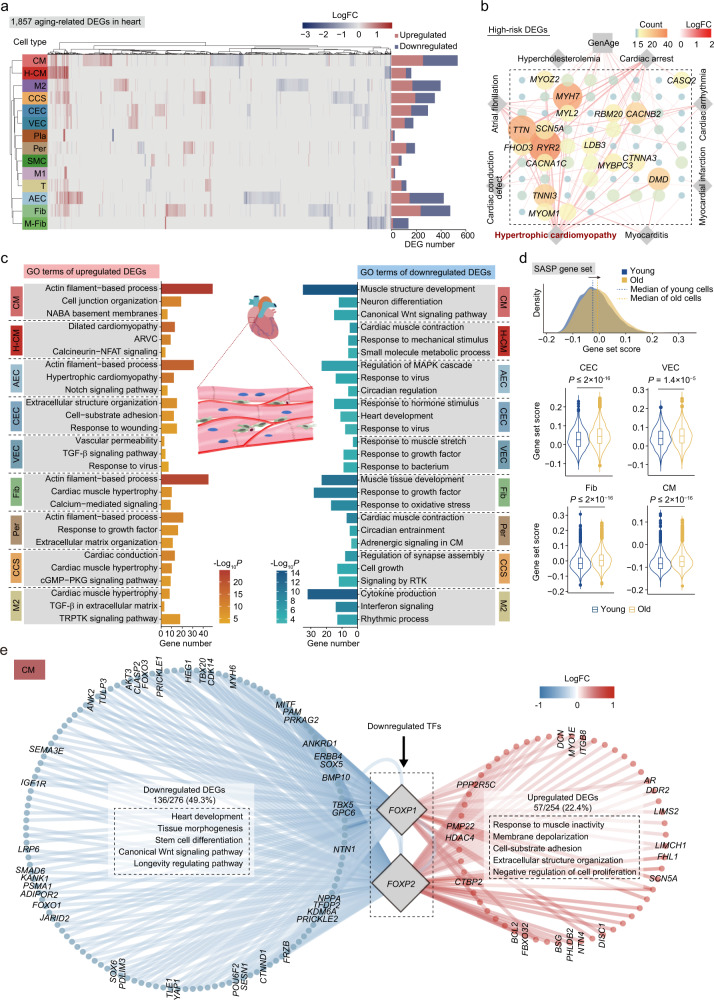
Age-related transcriptional alterations in various cell types of monkey heart. **a** Left, heatmap showing the DEGs during aging across different cell types in monkey heart. Right, bar plot showing the numbers of DEGs across different cell types in monkey heart (|logFC| > 0.25, adjusted *P* value < 0.05). **b** Network plot showing the upregulated DEGs associated with GenAge database (https://genomics.senescence.info/genes/) and heart diseases database (https://www.malacards.org/, https://www.disgenet.org/home/). The node size (count) of genes indicates the number of cell types across databases in which this gene was differentially expressed with age. The color of connecting lines indicates the logFC. Genes with count > 5 are shown. **c** Diagram showing the enrichment of GO terms (Biological Process) or pathways of nine cell types with the highest numbers of DEGs (|logFC| > 0.25, adjusted *P* value  < 0.05) in monkey heart. ARVC, arrhythmogenic right ventricular cardiomyopathy; TRPTK, transmembrane receptor protein tyrosine kinase; CM, cardiomyocyte; RTK, receptor tyrosine kinase. **d** Top, ridge plot showing the shift of SASP gene set score with age in all cells from monkey heart. Bottom, violin plots showing the cell types in which the SASP gene set score is significantly increased with age in monkey heart. **e** Network plot showing the differentially expressed *FOXP1* and *FOXP2* target genes (|logFC| > 0.25, adjusted *P* value < 0.05) in CMs. The enriched GO terms (Biological Process) for upregulated or downregulated target genes are shown in the rectangular boxes with dash lines.

To start exploring cell type-specific molecular mechanisms in aged heart, we identified a total of 1857 DEGs (|logFC| > 0.25, adjusted *P* value < 0.05) with differential expression in at least one cell type in aged versus young hearts (Fig. 3a; Supplementary information, Fig. S5a, Table S3). Among them, dozens of DEGs were also hotspot genes involved in heart diseases, especially hypertrophic cardiomyopathy (Fig. 3b; Supplementary information, Fig. S5a), reflecting a tight association between aging-related gene expression changes in the heart and the high incidence of cardiac diseases in the elderly.

To interpret the functional consequences of these gene expression patterns, we next analyzed annotated GO terms shared across cell types. Top-ranked upregulated pathways included “actin filament-based process”, “TGF-β signaling pathway”, and pathways indicative of hypertrophic and dilated cardiac remodeling (Fig. 3c; Supplementary information, Fig. S5b). Similar to what we observed in the aged lung, SASP-related genes were augmented in all major cell types of the heart, including endothelial cells, fibroblasts, and CMs (Fig. 3d), indicative of a similar chronic inflammation state in the aged heart. Pathways critical to cardiac structure and function, such as heart development, cardiac muscle contraction, and circadian clock (*PER1*, *PER2*, and *PER3*) were repressed in the aged heart (Fig. 3c; Supplementary information, Fig. S5b, and Table S3). The SCENIC analysis further pinpointed *FOXP1* and *FOXP2*, two highly homologous members of the forkhead TF family, as underlying the transcriptomic dynamics of aged CMs (Fig. 3e; Supplementary information, Fig. S5a, c and Table S4). The transcriptional network analysis predicted that *FOXP1* and *FOXP2* function broadly upstream of diverse gene programs involved in heart development, tissue morphogenesis, and longevity regulating pathways (Fig. 3e).

We also carefully assessed immune cells resident in the heart, especially macrophages and T cells, which constitute a large fraction of heart leukocyte populations. Cardiac anti-inflammatory macrophages (M2) that inhibit granulocyte activity of T cells^47^ and exhibit anti-inflammatory properties were the immune cell type most strongly affected by aging (Fig. 3a). One of the top upregulated genes identified in M2 macrophages was *HPGDS* (logFC = 1.00), encoding the catalytic enzyme for prostaglandin that generates inflammatory microenvironment (Supplementary information, Fig. S5d and Table S3).^48^ However, GO analysis showed that cytokine production and interferon signaling were repressed in aged M2 macrophages (Fig. 3c), implying a deregulated immune homeostasis and compromised anti-inflammatory function of aged M2 macrophages. In T cells, we found that in aged hearts, the most upregulated genes were *UNC5D* (logFC = 1.07) that augments chemokinesis and elicits a pro-inflammatory response,^49^ and *SH3RF2* (logFC = 0.92), a potential upstream activator of the NF-κB inflammatory pathway (Supplementary information, Fig. S5d and Table S3).^50^ Furthermore, the predicted frequency of global cell–cell communications was increased in aged hearts, especially between immune cells and pericytes (Supplementary information, Fig. S5e, f and Table S5). All our data point to the dysregulation of cardio immunology as a prominent feature of heart aging.

### Changes in putative target cell types of SARS-CoV-2 during primate lung aging

**Fig. 4:**
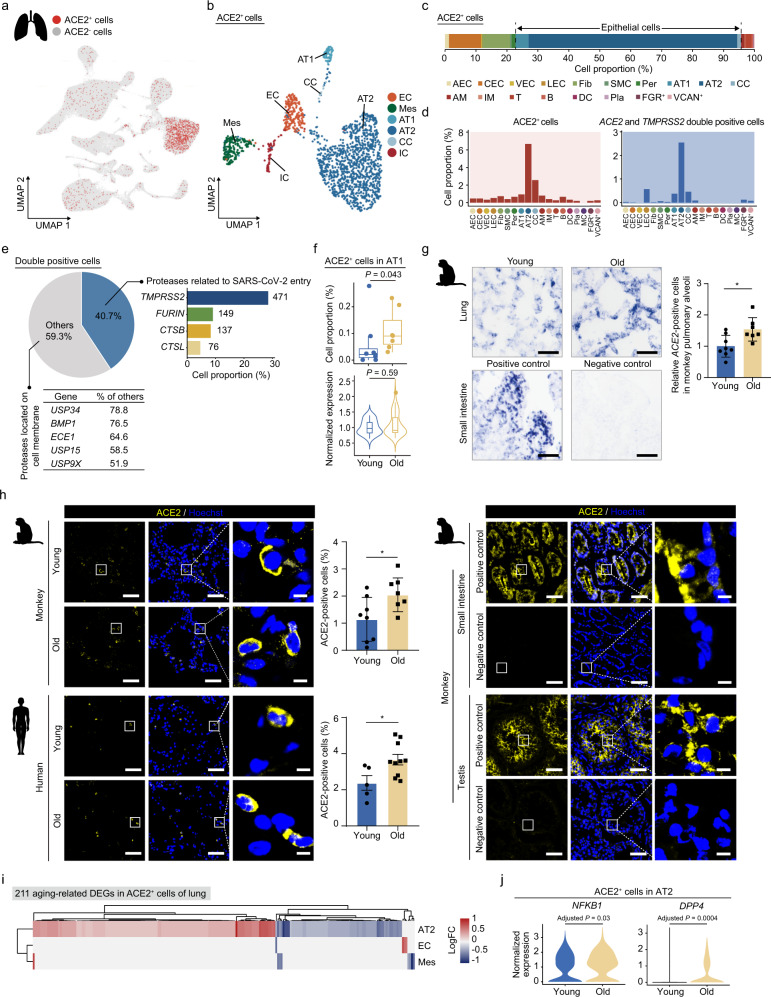
Changes in putative SARS-CoV-2 target cell types during monkey lung aging. **a** UMAP plot showing the ACE2^+^ cells in monkey lung. **b** UMAP plot showing the different ACE2^+^ cell types in monkey lung. EC, endothelial cell; Mes, mesenchymal cells (ciliated cells, fibroblasts, pericytes); CC, ciliated cell; IC, immune cell. **c** Bar plot showing the proportions of different ACE2^+^ cell types in monkey lung. The epithelial cells (AT1, AT2 and CC) are highlighted by dash lines. **d** Bar plots showing the proportions of ACE2^+^ cells (left) and *ACE2* and *TMPRSS2* double-positive cells (right) across different cell types in monkey lung. The asterisk denotes the cell type with the highest percentage of ACE2^+^ and *ACE2* and *TMPRSS2* double-positive cells, respectively. **e** Top left, pie plot showing the percentages of cells expressing genes associated with SARS-CoV-2 entry in ACE2^+^ cells of monkey lung. Top right, bar plot showing the percentages and numbers of cells expressing the indicated genes related to SARS-CoV-2 entry. Bottom, a table showing the percentages and numbers of cells expressing other membrane-bound proteases in ACE2^+^ cells. Only the five genes with the highest relative expression proportion are shown. **f** Box plots showing the proportion of ACE2^+^ cells and violin plot showing *ACE2* expression level in young and old AT1 cells. **g** Top, transverse sections of lung tissues from young and old monkeys were subjected to ISH with *ACE2* riboprobes. Representative ISH images are shown on the left; quantitative data are shown as means ± SEM on the right. Young, *n* = 8; old, *n* = 7 monkeys. *P < 0.05. Scale bar, 25 μm. Monkey small intestine section with *ACE2* riboprobes was used as positive control, and small intestine sections with *ACE2* reverse complementary riboprobes were used as negative control. The blue-purple signals were considered as ACE2-positive cells. **h** Immunofluorescence staining of ACE2 in young and old lung tissues from monkeys and humans, respectively. Quantitative data are shown as means ± SEM. Immunofluorescence staining of IgG and ACE2 in monkey small intestine and testis sections were used as negative and positive controls, respectively. Monkey, young, *n* = 8; old, *n* = 7 individuals. Human, young, *n* = 5; old, *n* = 10 individuals. Scale bars, 50 μm and 5 μm (zoomed-in image). *P < 0.05. **i** Heatmap showing DEGs across different cell types in ACE2^*+*^ cells of monkey lung. **j** Violin plots showing the expression levels of *NFKB1* and *DPP4* in ACE2^*+*^ AT2 across young and old groups.

To identify cell types potentially targeted by SARS-CoV-2, we analyzed cell type-specific expression patterns of *ACE2*, as well as *TMPRSS2*, *FURIN*, and other potential auxiliary proteins in our single-nucleus transcriptional lung and heart atlases from young and old cynomolgus monkeys (Fig. 4a; Supplementary information, Fig. S6a). In the lung, we found that *ACE2* was expressed in a fraction of AT2 (7%), AT1 (1%), ciliated cells (3%), fibroblasts (0.8%), pericytes (0.7%), and immune cells like AMs (0.8%), T cells (0.3%), B cells (0.7%) (Fig. 4b–d). We also found that a small fraction of endothelial cells expressed *ACE2*, 0.5% for both AECs and CECs (Fig. 4d). Most *ACE2* and *TMPRSS2* double-positive cells were detected in AT2 (2.6%) and AT1 (0.4%) (Fig. 4d). However, genes encoding one or more of other accessory proteases, including *FURIN*, *CTSB*, *CTSL* of cathepsin family members, and other membrane-anchored proteases, were mosaically co-expressed with *ACE2* in different cell types (Fig. 4e; Supplementary information, Fig. S6a). These data identified AT1 and AT2 as susceptible cell types for COVID-19 in the lung. Interestingly, the fraction of *ACE2*-positive AT1 cells was increased by ~2-fold in the aged lung (Fig. 4f), which was confirmed by RNA in situ hybridization (RNA-ISH) assay (Fig. 4g). More importantly, an increase in *ACE2*-positive cells around pulmonary alveoli was confirmed by immunostaining in both NHP and human lungs during aging (Fig. 4h; Supplementary information, Fig. S6b-d, and Table S1), implying that the fraction of cells more likely to be infected by SARS-CoV-2 at the surface of pulmonary alveoli increases with age.

Although the overall proportion of *ACE2*-positive AT2 cells remained unchanged with age, we identified *NFKB1* and its target gene *DPP4* as upregulated DEGs in *ACE2*-positive AT2 when comparing *ACE2*-positive cells between old and young lungs (Fig. 4i, j; Supplementary information, Fig. S6e). Of note, *DPP4*, previously reported as a factor downstream of NF-κB and increased in hypoxia-induced pulmonary hypertension,^51^ has come to the forefront as a candidate receptor for SARS-CoV-2 and is an established receptor for the Middle East Respiratory Syndrome Coronavirus (MERS-CoV).^52,53^ In the aged lung, we found that cell–cell interplays between the receptor TNFRSF1A (expressed by AT2) and its ligands TNFSF13 and GRN (potentially secreted by AM) were strengthened (Supplementary information, Fig. S6f and Table S5), a process known to be associated with constitutive NF-κB activation.^54^ These data suggest a potential regulatory loop, in which interaction with aberrant immune cells prompts AT2 respiratory epithelial cells to acquire an inflammation-prone state, in turn inducing expression of the coronavirus receptor.

### Age-related gene expression alterations in SARS-CoV-2 target cell types in the cardiovascular system

**Fig. 5:**
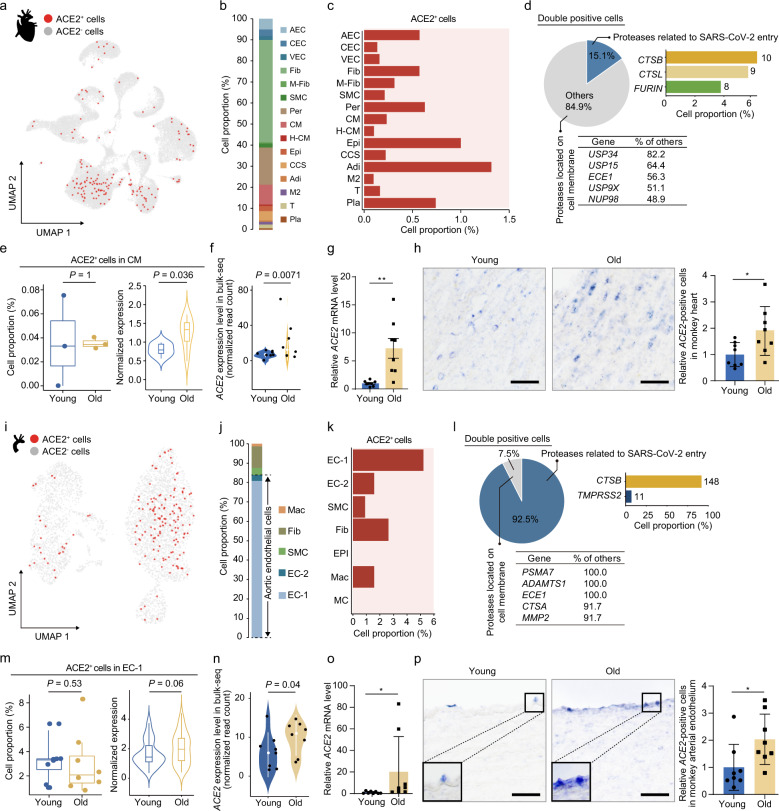
Age-related transcriptional alterations in SARS-CoV-2 target cell types in the cardiovascular system. **a** UMAP plot showing the ACE2^+^ cells in monkey heart. **b** Bar plot showing the proportions of ACE2^+^ cell types in monkey heart. **c** Bar plot showing percentages of ACE2^+^ cells across different cell types in the monkey heart. **d** Top left, pie plot showing the percentages of cells expressing genes associated with SARS-CoV-2 entry in ACE2^+^ cells of monkey heart. Top right, bar plot showing percentages and numbers of cells expressing the indicated genes related to SARS-CoV-2 entry. Bottom, the table showing the percentages and numbers of cells expressing other membrane-bound proteases in *ACE2*^+^ cells. Only the five genes with the highest relative expression proportion are shown. **e** Box plot showing the proportion of ACE2^+^ cells and violin plot showing *ACE2* expression level in young and old CMs. **f** Violin plot showing the *ACE2* expression levels in bulk RNA-seq analysis of monkey heart. **g** Transcript levels of *ACE2* in young and old monkey hearts quantified by RT-qPCR. Young, *n* = 8; old, *n* = 8 monkeys. The data are shown as means ± SEM. **P < 0.01. **h** Transverse sections of heart tissues from young and old monkeys were subjected to ISH with *ACE2* riboprobes. Representative ISH images are shown on the left; quantitative data are shown as means ± SEM on the right. Young, *n* = 8; old, *n* = 8 monkeys. *P < 0.05. Scale bar, 25 μm. The blue-purple signals were considered as *ACE2*-positive cells. **i** UMAP plot showing the ACE2^+^ cells in monkey aorta^.^ **j** Bar plot showing the proportions of ACE2^+^ cell types in monkey aorta. **k** Bar plot showing the percentages of ACE2^+^ cells across different cell types in monkey aorta. **l** Top left, pie plot showing the percentages of cells expressing genes associated with SARS-CoV-2 entry in ACE2^+^ cells of monkey aorta. Top right, bar plot showing the percentages and numbers of cells expressing the indicated genes related to SARS-CoV-2 entry. Bottom, a table showing the percentages and numbers of cells expressing other membrane-bound proteases in ACE2^+^ cells. Only the five genes with the highest relative expression proportion are shown. **m** Box plot showing the proportion of ACE2^*+*^ cells in EC-1 and violin plot showing the *ACE2* expression levels in ACE2^+^ cells. **n** Violin plot showing the *ACE2* expression levels in bulk RNA-seq analysis of monkey aorta between young and old groups. **o** Transcript levels of *ACE2* in young and old monkey aorta quantified by RT-qPCR. Young, *n* = 8; old, *n* = 8 monkeys. The data are shown as means ± SEM. *P < 0.05. **p** Transverse sections of aorta tissues from young and old monkeys subjected to ISH with *ACE2* riboprobes. Representative ISH images are shown on the left; quantitative data are shown as means ± SEM on the right. Young, *n* = 8; old, *n* = 8 monkeys. *P < 0.05. Scale bar, 25 μm. Zoom-in views of the region highlighted by dashed lines are shown in the left corner. The blue-purple signals were considered as *ACE2*-positive cells.

Alarming findings report that COVID-19 causes pervasive endotheliitis and blood clots, and viral particles have been detected in endothelial cells of COVID-19 victims.^55^ Whether and how SARS-CoV-2 attacks the cardiovascular system, however, remains incompletely understood. In heart, we found that *ACE2* was expressed mainly in a fraction of AECs (0.6%), fibroblasts (0.6%), pericytes (0.6%), SMCs (0.2%), and CMs (0.2%) (Fig. 5a–c). We were not able to detect *ACE2* and *TMPRSS2* double-positive cells in the heart. However, we found that *ACE2* was co-expressed with *FURIN*, *CTSB*, *CTSL*, and other protease genes (Fig. 5d; Supplementary information, Fig. S7a). Unlike the lung, the number of *ACE2*-positive cells in each cardiac cell type was not discernibly increased during aging (Fig. 5e; Supplementary information, Fig. S7b). Instead, the expression level of *ACE2* was upregulated in CMs during cardiac aging (Fig. 5e; Supplementary information, Fig. S7b). We also observed increased *ACE2* transcript and protein levels in the aged heart by bulk RNA sequencing (RNA-seq), quantitative real-time PCR (RT-qPCR), RNA-ISH, and immunostaining with ACE2 antibody (Fig. 5f–h; Supplementary information, Fig. S7c, d).

In a separate study using the same group of young and old cynomolgus monkeys, we recently depicted a single-cell transcriptomic landscape of aortic arteries using modified single-cell tagged reverse transcription (STRT) technique.^31^ Here, via in-depth analysis of the expression patterns of SARS-CoV-2 receptors in 4383 high-quality aortal wall cells, including endothelial cells (EC-1, EC-2), SMCs and adventitial fibroblasts (Fib) from different tunica layers (Supplementary information, Fig. S7e), we found that most *ACE2*-positive cells were present in endothelial cells (~5%), while a small fraction was detected in SMCs (1%) and fibroblasts (2%) (Fig. 5i-l; Supplementary information, Fig. S7f). The presence of *ACE2*-positive cells in artery endothelial cells raises the possibility that the vascular system could be directly invaded by SARS-CoV-2. Although the composition of *ACE2*-positive cells was unchanged during vascular aging (Fig. 5m; Supplementary information, Fig. S7g), endothelial cells from aged monkey aorta expressed higher levels of *ACE2* than their young counterparts (*P* value = 0.06) (Fig. 5m). Bulk RNA-seq with a high depth (*P* value = 0.04) and RT-qPCR (*P* value = 0.003) analyses of old versus young aorta also identified increased *ACE2* transcript level with age (Fig. 5n, o). RNA-ISH assays further validated the increased *ACE2* expression in aged arterial endothelium (Fig. 5p). In addition, top upregulated genes in aged *ACE2*-positive endothelial cells included ceruloplasmin (*CP*), the increase of which is associated with age-related chronic degenerative diseases, and proposed as a prognostic marker for recurrent infections (Supplementary information, Fig. S7h).^56,57^ These data pinpoint an upregulation of *ACE2* expression in aged primate vasculature, which may be associated with increased susceptibility of aged arteries to coronavirus infection.

### IL7 induces *ACE2* expression in HAECs

**Fig. 6:**
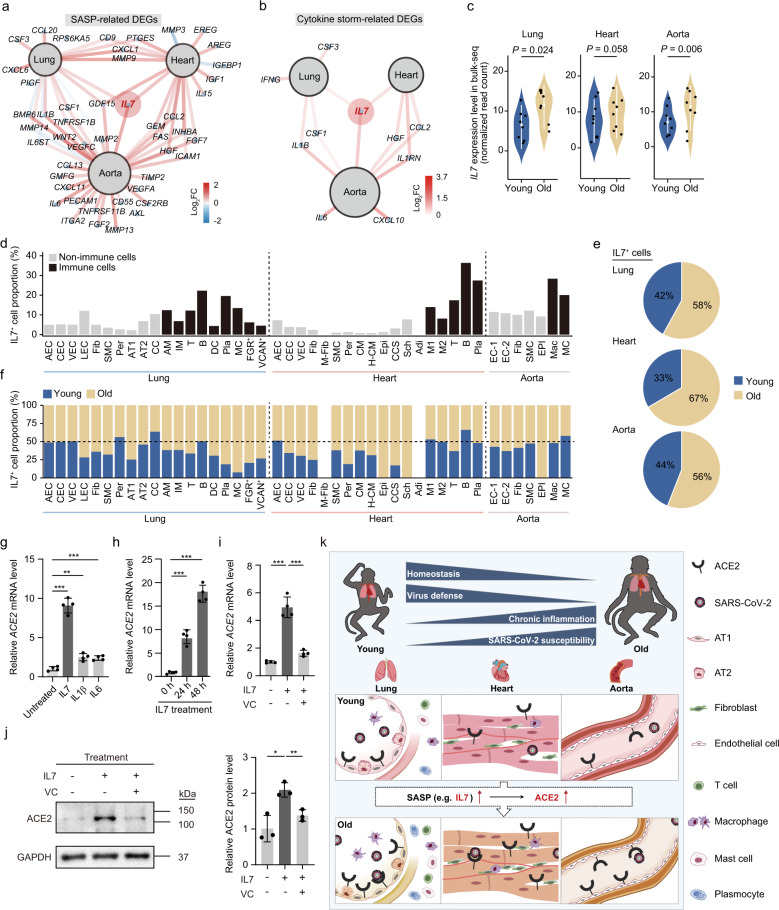
IL7 treatment stimulates ACE2 expression in human endothelial cells. **a** Network plot showing the DEGs related to SASP based on the bulk RNA-seq analysis of monkey lung, heart, and aorta. The color of connecting lines indicates the log_2_FC. Genes with *P* < 0.06 are shown. **b** Network plot showing the DEGs related to cytokine storm based on the bulk RNA-seq analysis of monkey lung, heart, and aorta. The color of connecting lines indicates the log_2_FC. Genes with *P* < 0.06 are shown. **c** Violin plots showing the transcript levels of *IL7* in monkey lung, heart, and aorta from bulk RNA-seq analysis. **d** Bar plots showing the proportions of IL7^+^ cells across different cell types in monkey lung, heart, and aorta. **e** Pie plots showing the total IL7^+^ cell proportions between young and old groups in monkey lung, heart, and aorta. **f** Bar plots showing the IL7^+^ cell proportions between young and old groups across different cell types in monkey lung, heart, and aorta. **g** RT-qPCR showing the upregulation of *ACE2* expression after a 6-h treatment with IL7 (10 ng/mL), IL1β (10 ng/mL) and IL6 (25 ng/mL) in HAECs (passage 1). The data are shown as means ± SEM, *n* = 4 experimental repeats, and the experiment was independently repeated three times with similar results. **P < 0.01, ***P < 0.001. **h** RT-qPCR showing the upregulation of *ACE2* expression after treatment with IL7 (10 ng/mL) at different time points in HAECs (passage 2). The data are shown as means ± SEM, *n* = 4 experimental repeats, and the experime*n*t was independently repeated three times with similar results. ***P < 0.001. **i** RT-qPCR detection of *ACE2* expression in HAECs (passage 4) after treatment with IL7 in the presence or absence of vitamin C. Quantitative data are shown as means ± SEM. *n* = 4. ***P < 0.001. **j** Western blot analysis of ACE2 expression in HAECs after treatment with IL7 (10 ng/mL) and vitamin C (280 μM). GAPDH was used as a loading control. Quantitative data are shown as means ± SEM. *n* = 3 experimental repeats. *P < 0.05; **P < 0.01. **k** Schematic illustration of the functional decay and increased susceptibility to SARS-CoV-2 with age revealed by a transcriptomic atlas of aged primate cardiopulmonary system. SASP secretion increases (such as IL7) with age, thus promoting an inflammatory environment and inducing ACE2 expression, which may result in increased susceptibility to SARS-CoV-2.

Finally, we sought to explore what factors associated with aging might mediate ACE2 upregulation in primate tissues. We focused our attention on IL7, as our bulk RNA-seq analysis had revealed that IL7 was not only commonly upregulated in the aged lung (*P* value = 0.024), heart (*P* value = 0.058), and aorta (*P* value = 0.006) (Fig. 6a–c; Supplementary information, Fig. S8), but was also among the cytokines that drive the immune overreaction known as cytokine storm in patients with COVID-19 (Fig. 6a–c; Supplementary information, Table S6).^9,58–60^ snRNA-seq demonstrated that IL7 was pervasively expressed in several cell types in monkey lung and cardiovascular system (Fig. 6d), and the proportion of IL7-expressing cells was increased in several aged monkey cell types, including AT1, CMs, and VECs (Fig. 6e, f). Immunofluorescence staining with anti-IL7 antibody confirmed increased IL7 protein levels in aged lung and vascular tissues (Supplementary information, Fig. S8e, f). To test whether IL7 is capable of inducing *ACE2* expression, we treated HAECs with IL7 and other SASP factors, including IL1β and IL6 (Fig. 6g; Supplementary information, Fig. S8e). Notably, IL7 treatment induced a greater increase in *ACE2* expression compared to IL1β and IL6 treatments (Fig. 6g). We also found that IL7 stimulation upregulated *ACE2* expression over the time period tested, with the highest outputs recorded at two days after drug treatment (Fig. 6h). In addition, IL7 treatment of HAECs markedly upregulated ACE2 protein level by western blot analysis (Supplementary information, Fig. S9a). Therefore, these results suggest that IL7 plays a critical role in upregulating *ACE2* expression during primate aging, at least in HAECs.

Furthermore, the bioinformatic analysis identified *RELA* (encoding a subunit of NF-κB) as one of the top genes upregulated in *ACE2*-positive cells compared to those in *ACE2*-negative cells (Supplementary information, Fig. S9b). Transcriptomic analysis revealed upregulated NF-κB target genes in aged lung and cardiovascular tissues (Supplementary information, Fig. S9c). We then generated CRISPR/Cas9-mediated *RELA*-knockout human vascular endothelial cells (hVECs) and found that IL7 failed to induce the expression of *ACE2* in the absence of RelA (Supplementary information, Fig. S9d, e), suggesting a pivotal role of NF-κB in mediating this process. As geroprotective agents like vitamin C have been reported to inhibit NF-κB activation,^61,62^ we tested the effect of vitamin C on *ACE2* expression. We found that pretreatment of vitamin C blunted IL7-induced *ACE2* expression in HAECs (Fig. 6i, j; Supplementary information, Fig. S9e). Altogether, these findings add new insights into ACE2 regulation in aged VECs and suggest that geroprotective strategies may reduce COVID-19 severity in the elderly.

## Discussion

In this study, we analyzed the single-nucleus transcriptomic maps of aged primate respiratory and cardiovascular tissues to understand the cellular and molecular basis for their vulnerabilities to age-related diseases and COVID-19 (Fig. 6k). Overall, we found that critical features of primate pulmonary and cardiovascular aging included compromised tissue homeostasis and viral defensibility as well as activation of inflammatory responses (Fig. 6k). Strikingly, we discovered that expression of ACE2, the cellular entry receptor for SARS-CoV-2, was upregulated in the epithelial barrier layer of the pulmonary alveolus, in CMs, and in VECs with age (Fig. 6k). At the molecular level, we found that age-related IL7 upregulation robustly induced ACE2 expression in VECs (Fig. 6k). In addition, vitamin C potently inhibited IL7-induced ACE2 expression. Altogether, we here depict the first transcriptomic atlas of the aged primate cardiopulmonary system, which provides mechanistic insights into how aging-related inflammation and susceptibility to microorganismal infection are interconnected to increase the vulnerability of older human to COVID-19.

snRNA-seq technology has recently been employed by us and others to reveal changes in cell composition and cell type-specific gene expression accompanying aging.^17^ From an advantaged perspective, snRNA-seq provides less cell type composition bias than traditional scRNA-seq, especially for tissue types that are challenging to dissociate into single cells.^17,63^ Recently, several studies report SARS-CoV-2 entry factors using single-cell transcriptomic profiling of adult lung and airway tissues.^23,24,64,65^ Reanalyzing scRNA-seq data of smoking individuals revealed that cigarette smoke causes a dose-dependent increase of ACE2 in rodent and human lungs.^66^ However, the alteration of ACE2 expression during aging is still controversial.^66–68^ In this study, we present the first comparative single-nucleus/single-cell sequencing atlas of old versus young primate lung and cardiovascular tissues, revealing that ACE2 expression was upregulated in a both age-dependent and cell type-specific manner. The aging-associated upregulation of ACE2 was further confirmed by multiple assays, such as in-depth bulk RNA-seq, RT-qPCR, RNA-ISH, and immunostaining in various tissues. These results may eventually provide a scientific explanation for the higher risk and greater severity observed in older people for COVID-19 and better treatment options. Our analysis of viral susceptibility relied primarily on the current knowledge that ACE2 is a determinant of viral entry. However, it is possible but unknown whether SARS-CoV-2 uses other receptors or co-receptors for cell entry. For instance, the recent finding that TMPRSS2-negative cells may use cathepsin B/L7 for alternative viral entry complicates the identification of SARS-CoV-2 target cells.^23^ By tracing the viral infection process at consecutive infection stages in primates via single-cell transcriptomic analysis, we stand to better understand the host–virus relationship, which might help find potential druggable targets.

As a feature of aging, low-grade chronic inflammation not only influences the pace of aging but also affects the onset of aging-related diseases.^16,69,70^ Similar to low-grade inflammation, cytokine storm in COVID-19 pathogenesis also involves the production and secretion of proinflammatory cytokines.^71,72^ In addition, we have recently reported that SARS-CoV-2 promotes age-associated immune cell polarization along with the expression of pro-inflammatory and pro-senescence genes including IL1β and type I interferon (IFN-α).^73^ Among them, IL1β and IFN-α were recently reported to be able to stimulate ACE2 expression in cultured human lung epithelial cells.^28,74^ For the first time, our data show that IL7, an aging-associated inflammatory cytokine, robustly upregulates ACE2 expression in HAECs. These data, together with the notion that both IL1β and IFN-α themselves are key SASP factors,^69,73,75,76^ strongly support a scientific scenario where systemic inflammation strengthens the severity of COVID-19 in the elderly population, and is, therefore, a potential intervention target for COVID-19 treatment.

Our study suggests several preventive strategies and potential therapeutic targets against COVID-19. Firstly, our data indicate a tight association between hypoxia responses and induction of inflammation in aged primate lung. Such a vicious underlying association is likely exacerbated upon viral infection, given that patients with COVID-19 pneumonia are already suffering from hypoxia even before the onset of severe symptoms.^77–79^ In fact, early or advanced intervention to improve oxygen saturation has been proved to lower the probability of a sudden crush in COVID-19 patients.^80^ Secondly, other possible targets may be the SASP factors, their upstream production regulators, or their downstream effector molecules. In addition to previously reported SASP factors such as IL1β and IFN-α,^81^ we here identified IL7 as an upstream inducer for ACE2 production in VECs. Hence, blocking IL7 signaling may help lower systemic or local ACE2 levels and alleviate SARS-CoV-2 symptoms, providing a scientific rationale for testing anti-IL7 antibodies or IL7 blockers as a treatment for severe COVID-19 patients with advanced age.^82^ Lastly, repurposing of geroprotective agents to COVID-19 treatment can be a candidate prevention/treatment strategy, especially for vulnerable aged patients. Consistent with our observations that treatment with vitamin C blunted IL7-induced ACE2 expression in HAECs, a clinical trial has recently been launched to test the possible effect of high-dose vitamin C in patients with COVID-19.^83^ Therefore, attenuating senescence may indeed provide a practical solution to reduce systemic inflammation and increase the aging body’s inherent resistance to viruses.

## Materials and methods

### Animals

The use of cynomolgus monkeys in this study has been approved by the Ethics Review Committee of the Institute of Zoology of the Chinese Academy of Sciences. Eight young monkeys (4–6 years old) and eight old monkeys (18–21 years old) originating from Southeast Asia were raised at 25 °C, 12-h light and dark cycle at a certified Primate Research Center in Beijing (Xieerxin Biology Resource).^31,84,85^ All animals used were confirmed to have no clinical, experimental, or pregnancy history before the start of the experiment. Detailed information of the animals used is shown in Supplementary information, Table S1.

### Tissue sampling

Organs and tissues were harvested from anesthetized monkeys perfused with physiological saline. The lungs from 15 of the 16 animals were collected after the tissues turned white (the lung from an old male individual was excluded due to limited technical experience at the beginning of the experiment). Lung tissues were dissected, and attached fat tissues were carefully removed. Left lung tissues were then kept in 4% paraformaldehyde (PFA)^86^ at 4 °C for histological analyses, and right lung tissues were frozen in liquid nitrogen until nucleus isolation for sequencing analysis and analyses of protein and RNA. Hearts were dissected on ice and divided into five parts: the right atrium, left atrium, right ventricle, left ventricle, and interventricular septum. One third of each part was kept in 4% PFA at 4 °C for histological analyses, and the remaining tissues were frozen in liquid nitrogen. Due to time and budget restriction, only the right atria of six male animals (3 young monkeys, 3 old monkeys) were subjected to nucleus isolation and further sequencing analysis. We would like to note that the aortic tissues used in the combined analysis here were obtained from the same 16 individual cynomolgus monkeys of which the phenotypic and sequencing data on aortic tissues have been published.^31^

For human lung aging study, human paratumor tissues were collected under the approval of the Research Ethics Committee of the First Hospital of Kunming Medical University. Fifteen patients diagnosed with primary lung cancer, including five young individuals aged 29–39 years (2 females and 3 males) and ten old individuals aged 66–77 years (4 females and 6 males) were randomly selected and included in this study (Supplementary information, Table S1). Paratumor tissue samples were collected from normal tissues 3 cm away from the tumor edge, and all the samples were confirmed by pathological examinations before being subjected to immunofluorescence staining with the anti-ACE2 antibody.

### H&E staining

H&E staining was performed as previously described.^31,85^ Tissues were preserved in 4% PFA following dehydration in a graded series of alcohols (70%–100%). Next, the tissues were paraffin-embedded and sectioned at a 5-μm thickness using a rotary microtome. Sections were placed on glass microscope slides, dried at 56 °C for 2 h, and stored at room temperature (RT) for later use. For H&E staining, sections were deparaffinized in xylene, rehydrated in gradient alcohols (100%, 100%, 95%, 80%, 75%), and then briefly rinsed in distilled water. Tissue sections were incubated with hematoxylin solution until the desired degree of staining (Servicebio, China), and rinsed in running water to remove excess hematoxylin. Sections were then differentiated in 1% acid alcohol for 1 s and rinsed in running water for 1 min. Lastly, the sections were stained with eosin to the desired shade of pink, dehydrated in gradient ethanol and xylene, and mounted with cytoseal-60 (Stephens Scientific). The depth of cardiac fatty infiltration was evaluated by the distance from epicardium to the furthest infiltrated adipocytes in the cardiac tissues in young and old monkey hearts.

### Masson’s trichrome staining

Masson’s trichrome staining was performed as previously described.^35^ Paraffin-embedded sections were deparaffinized in xylene and rehydrated in gradient ethanol (100%, 95%, 70%). After rinsing with distilled water, sections were stained with potassium dichromate solution at RT overnight and rinsed in running water for 5–10 min. Sections were then stained with iron hematoxylin working solution for 10 min, and rinsed in warm running water for 10 min, followed by staining with Ponceau-acid fuchsin solution for 5–10 min and rinsing in distilled water. Next, sections were differentiated in the phosphomolybdic-phosphotungstic acid solution for 10–15 min or until the tissues were no longer red, and directly stained (without rinse) in aniline blue solution for 5–10 min, followed by a brief rinse in distilled water and differentiation in 1% acetic acid solution for 2–5 min. After several washes in distilled water, sections were quickly dehydrated in 95% ethyl alcohol and absolute ethyl alcohol, cleared in xylene, and mounted with a resinous mounting medium.

### SA-β-Gal staining

SA-β-Gal staining was performed using a previously published protocol.^87,88^ In brief, OCT-embedded, snap-frozen, unfixed primate tissues were cryosectioned at a thickness of 30 μm with a Leica CM3050S cryomicrotome, collected on Superfrost Plus microslides (VWR) and kept at –80 °C until use. For SA-β-Gal staining, sections were thawed at RT and rinsed in PBS, fixed in 2% formaldehyde and 0.2% glutaraldehyde at RT for 5 min and stained with freshly prepared staining solution at 37 °C (3 days for the lung and one week for the heart) (X-gal was purchased from Amresco; all the other reagents were from Sigma-Aldrich). Images were taken with an Olympus CKX41 microscope, and the percentages of SA-β-Gal**-**positive cells were quantified using ImageJ.

### Oil Red O staining

Oil Red O staining was performed using a previously published protocol.^89^ Briefly, OCT-embedded, snap-frozen primate tissues were cryosectioned at a thickness of 10 μm with a Leica CM3050S cryomicrotome. Frozen sections were stained in freshly prepared Oil Red O staining solution (Sigma-Aldrich) at 60 °C for 8–10 min, washed in running tap water, and counterstained with hematoxylin. Images were taken with a Leica Aperio CS2 system, and the percentages of Oil Red O-positive cells were quantified using ImageJ.

### RNA-ISH

In brief, lung, right atrial, and aortic tissues were fixed with 4% PFA at 4 °C overnight, infused in 25% sucrose solution, and embedded in OCT compound (Thermo Fisher Scientific) for cryoprotection. OCT-embedded tissues were cut into slices at a thickness of 18 μm for ISH. Primers used for the cloning of *ACE2* fragments from monkey cDNA and subsequent RNA probe labeling are listed in Supplementary information, Table S7. DIG-labeled RNA probes were transcribed by T7 and T3 RNA polymerases using DIG RNA Labeling Mix (Roche Diagnostics). ISH was performed following a slightly modified protocol, as previously described.^90^ The blue-purple signals were considered as *ACE2*-positive cells.

### Immunofluorescence staining

Immunofluorescence staining was performed as previously described.^85^ Paraffin-embedded sections were deparaffinized in xylene and rehydrated through gradient alcohol (100%, 100%, 90%, 80%, 70%, 50%). After rinsing in distilled water, sections were microwaved in 10 mM sodium citrate buffer (pH 6.0) three times for 2 min each. Upon cooling down to RT, sections were rinsed three times in PBS, permeabilized with 0.4% Triton X-100 in PBS for 30 min and rinsed again in PBS three times. Sections were then incubated with blocking buffer (10% donkey serum in PBS) at RT for 1 h, followed by incubation with primary antibodies overnight at 4 °C and fluorescence-labeled secondary antibodies at RT for 1 h. Nuclei were counterstained with Hoechst 33342 (Thermo Fisher Scientific) before the sections were mounted in VECTERSHIELD anti-fading mounting medium (Vector Laboratories, h-1000). Images were obtained using a confocal laser scanning microscope (Leica TCS SP5 II). Antibodies used for immunofluorescence analysis are listed in Supplementary information, Table S7.

### Immunohistochemistry staining

Immunohistochemistry staining was performed as previously described.^88^ Paraffin-embedded sections were deparaffinized and rehydrated, followed by antigen retrieval by microwaving in 10 mM sodium citrate buffer (pH 6.0) three times for 2 min each. After being cooled down to RT, sections were rinsed in PBS three times and incubated with 3% H_2_O_2_ for 10 min to inactivate endogenous peroxidase. Sections were then blocked with 10% donkey serum in PBS for 1 h and incubated with primary antibodies at 4 °C overnight. The next day, sections were incubated with HRP-conjugated secondary antibodies at RT for 1 h, followed by colorimetric detection using DAB and counterstaining with hematoxylin. Finally, sections were dehydrated in a series of graded alcohols (50%, 70%, 80%, 90%, 100%, and 100%) and xylene before being mounted in the neutral resinous mounting medium. Antibodies used for immunohistochemistry staining are listed in Supplementary information, Table S7.

### RNA isolation and RT-qPCR

Tissue samples were snap-frozen in cryotubes submerged in liquid nitrogen. Total RNA was extracted using TRIzol (Life Technologies, 15596018) according to the manufacturer’s protocol. cDNAs were then reverse transcribed using 2 μg of total RNA as the template with the GoScript™ reverse transcription system (Promega). RT-qPCR was conducted using the iTaq Universal SYBR Green SuperMix (Bio-Rad) on a CFX384 real-time PCR system (Bio-Rad). The relative mRNA expression level of each gene was normalized to GAPDH expression, calculated using the ∆∆Cq method. At least three independent samples were used for RT-qPCR assay, and the differences between the two groups were analyzed by an independent-sample *t*-test. Primers used are listed in Supplementary information, Table S7.

### Generation of *RELA*^–/–^ hVECs

Both *RELA*^+/+^ and *RELA*^**–/–**^ human embryonic stem cells (hESCs) were differentiated into hVECs.^31,91–94^ In brief, hESCs were picked into 6-well plate coated with Matrigel and cultured with mTeSR on day 0. hESCs were washed with IMDM (Gibco) and cultured with EGM-2 medium (Lonza) supplemented with 25 ng/mL BMP4 (R&D), 3 μM CHIR99021 (Selleck), 3 μM IWP2 (Selleck) and 4 ng/mL FGF2 (JPC) on day 1 through day 3. The medium was then replaced by EGM-2 medium supplemented with 50 ng/mL VEGF (HumanZyme), 10 ng/mL IL6, and 20 ng/mL FGF2 (JPC) for another 3 days. CD201-PE (Biolegend, 351904, 1:300) and CD34-FITC (BD biosciences, 555821, 1:100) were used to sort and purify hVECs.

### Cell culture and IL7 stimulation of HAECs

HAECs were cultured in 6-well plates (Corning Incorporated, 3516) with EGM-2 medium to reach ~80% confluence. Human recombinant IL7 was incubated for 6 h, 24 h, and 48 h at a final concentration of 10 ng/mL. Human recombinant IL1β and IL6 were incubated for 6 h at a final concentration of 10 ng/mL and 25 ng/mL, respectively. The cells were then collected, and total RNA was extracted using TRIzol reagent and converted to cDNA using GoScript Reverse Transcription System for subsequent RT-qPCR analysis. To test the effect of vitamin C on IL7-induced *ACE2* expression, HAECs (passage 4) were cultured to ~40% confluence and treated with vitamin C at 280 μM for 48 h, followed by incubation with both human recombinant IL7 (10 ng/mL) and vitamin C for another 48 h. Cells were collected for RNA extraction and RT-qPCR was conducted to examine *ACE2* expression level. Cell lines, cell culture medium, and cytokines used are listed in Supplementary information, Table S7.

### Transfection and western blot

HEK293T cells were transfected with plasmid containing the full-length *ACE2* gene using Lipofectamine 3000 (Thermo, L3000015). pcDNA3.1-ACE2–3× FLAG was purchased from Zoman Biotechnology (ZK0752), and pcDNA3.1-ACE2-GFP was kindly provided by Prof. Chenyu Jiang.^95^ Transfected HEK293T pellets were lysed with RIPA buffer (0.1% SDS, 50 mM Tris-HCl, pH 7.5, 1% NP-40, 1 mM EDTA and 150 mM NaCl) added with protease inhibitor mixture (Roche) on ice for 30 min. The samples were centrifuged at 13,000 rpm at 4 °C for 30 min, and the supernatant was collected and stored at –80 °C for later use. Protein concentration was quantified using a BCA kit (Dingguochangsheng Biotechnology, BCA-02). 20 μg of protein lysate was separated by SDS-PAGE and then transferred to a PVDF membrane (Millipore). After incubation with primary antibodies at 4 °C overnight and with HRP-conjugated secondary antibodies at RT for 1 h, blots were visualized using the ChemiDoc XRS system (Bio-Rad, Hercules, CA, USA). Antibodies used for western blot are listed in Supplementary information, Table S7. In particular, western blot of ACE2 was performed in HEK293T cells transfected with FLAG-ACE2 and ACE2-GFP plasmids. The specificity of the ACE2 protein band verified the reliability of the anti-ACE2 antibody used for immunostaining.

### Nucleus isolation and snRNA-seq on the 10× Genomics platform

Nucleus isolation was performed using a previously published protocol.^96^ In brief, frozen primate tissues were pestled and solubilized in 1.5 mL lysis buffer containing 250 mM sucrose, 25 mM KCl, 5 mM MgCl_2_, 10 mM Tris buffer, 1 μM DTT, 1× protease inhibitor, 0.4 U/μL RNaseIn, 0.2 U/μL Superasin, 0.1% Triton X-100, 1 μM propidium iodide (PI), and 10 ng/mL Hoechst 33342 in Nuclease-Free water. Samples were filtered through a 40-micron cell strainer (BD Falcon), centrifuged at 1000× *g* for 8 min at 4 °C, and resuspended in PBS supplemented with 0.3% BSA, 0.4 U/μL RNaseIn and 0.2 U/μL Superasin. Nuclei were sorted for both Hoechst 33342 and PI using FACS (BD Influx) and counted with a dual-fluorescence cell counter (Luna-FL^TM^, Logos Biosystems). Mononuclear capture was conducted with a 10× Genomics single-cell 3’ system. Approximately 5,000 nuclei were captured for each sample following the standard 10× capture and library preparation protocol (10× Genomics) and then sequenced in a NovaSeq 6000 sequencing system (Illumina, 20012866).

### Bulk RNA-seq library construction and sequencing

Total RNAs of individual cynomolgus monkey lung, heart, and aortic tissues were extracted using TRIzol. RNA quality control, library construction, and high-throughput sequencing were performed for each sample by Novogene Bioinformatics Technology Co. Ltd. Briefly, sequencing libraries were prepared using NEBNext^®^ UltraTM RNA Library Prep Kit for Illumina^®^ (NEB, USA) and individually indexed. The resultant libraries were analyzed on an Illumina paired-end sequencing platform by 150-bp read length by Novogene Bioinformatics Technology Co. Ltd.

### Processing of snRNA-seq data

Sequences from the NovaSeq analysis were demultiplexed using bcl2fastq (version 2.20.0.422) to convert BCL to FASTQ files. Pre-mRNA reference of *Macaca fascicularis* (version Macaca_fascicularis_5.0 sourced from Ensemble) was created following the Cell Ranger (version 3.1.0) protocol (https://support.10xgenomics.com/single-cell-gene-expression/software/pipelines/latest/advanced/references). Raw snRNA-seq data were demultiplexed and mapped to the pre-mRNA reference. Gene count was calculated by Cell Ranger with the default parameters. The expression matrices were then obtained for downstream analyses.

### Filtering of low-quality cells

The output expression matrix from Cell Ranger was calculated with Seurat (version 3.1.3).^97^ Cells with fewer than 200 genes or with a mitochondrial gene ratio of more than 5% were regarded as low-quality cells and therefore excluded. Possible doublets were detected using DoubletFinder (version 2.0.2).^98^ The mean-variance normalized bimodality coefficient (BCMVN) was calculated to determine the neighborhood size (pK) by each sample, and the number of artificial doublets (pN) was set to 0.25 as the recommended parameters. Defined by using the “doubletFinder_v3” function, a total of 7531 and 2013 doublets were excluded from the lung and heart, respectively. In the end, a total of 109,609 cells for lung and 42,053 for heart with high quality were further analyzed.

### Clustering and identification of cell types

After doublet removal, data of each sample were normalized using the function “SCTransfrom” of Seurat. Features and anchors for downstream integration were selected with “PrepSCTIntegration” and “FindIntegrationAnchors”, ensuring that all necessary Pearson residuals have been calculated. After data integration and scaling, the principal component analysis was performed by the “RunPCA” function, and clustering was then conducted using the “FindClusters” function. Marker genes for each cluster were identified with the “FindAllMarkers” function, and only those with adjusted *P* values < 0.05 and |logFC| > 1 were regarded as marker genes. In addition, 2 clusters in the lung snRNA-seq data lacking significant expression of canonical marker genes and having relatively low gene numbers and high mitochondrial gene ratios compared to other clusters were excluded as low-quality cells. Cell types were identified according to the expression of canonical marker genes for each cluster (Supplementary information, Table S2).^31,67,85,99,100^ Dimensionality reduction was performed with the “RunUMAP” function and the resultant data were visualized with the “DimPlot” function.

### Analysis of DEGs from snRNA-seq data

Differential gene expression analysis was performed with the “FindMarkers” function of Seurat between old and young groups using the Wilcox test. Only those with adjusted *P* values < 0.05 and |logFC| > 0.25 were identified as DEGs. By the standard that any cell types with numbers of cells less than three in any individual would be excluded from DEG analysis, four cell types of the lung (dendritic cells, plasmocytes, mast cells, and *FGR*^+^ cells) and four cell types of the heart (Schwann cells, B cells, adipose cells, and epicardial cells) were excluded.

### Analysis of TF regulatory network

TF regulatory network analysis was performed using SCENIC workflow (version 1.1.2.2)^101^ with default parameters. hg19 TFs were downloaded using RcisTarget (version 1.6.0) as a reference. Gene regulatory networks were inferred with GENIE3 (version 1.6.0) based on the 1356 aging-related DEGs for lung and 1857 for heart. Enriched TF-binding motifs, predicted candidate target genes (regulons), and regulon activity were inferred by RcisTarget. The transcription regulatory network was visualized by Cytoscape (version 3.7.2).^102^

### GO and pathway enrichment analysis of DEGs

GO and pathway enrichment analysis of aging-related DEGs was performed by Metascape (http://metascape.org/gp/index.html) (version 3.5).^103^ Results were visualized with the ggplot2 R package (https://ggplot2.tidyverse.org/) (version 3.2.1).

### Re-clustering of subgroups of *ACE2*-positive cells in the lung

*ACE2*-positive cells were defined as cells with *ACE2* mRNA expression level > 0. After identifying the subgroups of *ACE2*-positive cells, the dataset was re-clustered as aforementioned with appropriate resolution.

### Gene set score analysis

Gene sets score for each input cell were calulated by the Seurat function “AddModuleScore”. Changes in the scores between young and old samples were analyzed using ggpubr package via Wilcox test (https://github.com/kassambara/ggpubr) (version 0.2.4).

### Cell–cell communication analysis

Cell–cell communication analysis was conducted with the snRNA-seq data by using the CellPhoneDB software (version 1.1.0) (www.cellphonedb.org).^104^ Only receptors and ligands expressed in >10% of cells of any type from old or young group were further evaluated, while a cell–cell communication was considered nonexistent if the ligand or the receptor was unmeasurable. Averaged expression of each ligand-receptor pair was analyzed between various cell types, and only those with *P* value < 0.01 were used for the prediction of cell–cell communication between any 2 cell types.

### Cumulative density distribution analysis of gene expression

The scRNA-seq data of EC-1 cells were retrieved based on the defined cell types in monkey aorta. Then, cells with *ACE2* expression levels higher than 0, and cells with *ACE2* expression levels equal to 0 were defined as ACE2^+^ cells and ACE2^–^ cells, respectively. For each gene, the difference in its mRNA expression levels between ACE2^+^ cells and ACE2^–^ cells was examined with Kolmogorov–Smirnov (K–S) test and indicated by the calculated *D* and *P* values. The results were plotted with ggplot2 package.

### Bulk RNA-seq data processing

RNA-seq data were processed as previously reported.^105^ Pair-end raw reads were trimmed using the TrimGalore software (version 0.4.5) (https://github.com/FelixKrueger/TrimGalore). The reads were mapped to the UCSC MacFas 5.0 genome using hisat2 (version 2.0.4) for monkey samples.^106^ The sam files were converted to bam files by SAMtools (version 1.6) with the parameter “-S -b -q 10”.^107^ Read counts for each gene were then calculated by HTSeq (version 0.11.0), with only high-quality mapped reads (score of mapping quality > 20) kept for further analysis.^108^ DEGs were identified using R package DESeq2 (version 1.22.2)^109^ with a cutoff *P* value < 0.05.

### NF-κB target gene analysis

To investigate the transcriptional changes of NF-κB target genes in aorta, heart, and lung aging, we obtained a list of NF-κB target genes from http://www.bu.edu/nf-kb/gene-resources/target-genes/. Then density plots were drawn to compare the expression levels (log_2_FC) of NF-κB target genes and other genes between old and young tissues (shown in Supplementary information, Fig. S9b). *P* values were evaluated by the two-sided Wilcoxon rank-sum test in R.

### Statistical analyses

All data were statistically analyzed using a two-tailed *t-*test to compare differences between different groups, assuming equal variance with PRISM software (GraphPad 6 Software). Asterisks are used as indicators for statistical differences for experimental data; *P* values are presented for bioinformatic analyses. *P* value < 0.05 was considered statistically significant. *P* < 0.05, *P* < 0.01 and *P* < 0.001, NS, not significant.

## Supplementary information

supplementary information, Fig S1

supplementary information, Fig S2

supplementary information, Fig S3

supplementary information, Fig S4

supplementary information, Fig S5

supplementary information, Fig S6

supplementary information, Fig S7

supplementary information, Fig S8

supplementary information, Fig S9

Supplementary information, Table S1

Supplementary information, Table S2

Supplementary information, Table S3

Supplementary information, Table S4

Supplementary information, Table S5

Supplementary information, Table S6

Supplementary information, Table S7

## Data Availability

The accession numbers for the raw snRNA-seq data reported in this paper in Genome Sequence Archive (GSA): CRA002577 (lung), CRA002689 (heart). The accession numbers for the raw bulk RNA-seq data in GSA: CRA002810.
