# Supplementary material for: Single-cell transcriptomic atlas of primate cardiopulmonary aging
Source: Cell Res. 2020 Sep 10;31(4):415–32. doi: 10.1038/s41422-020-00412-6 (PMC7483052; doi:10.1038/s41422-020-00412-6)
Supplement: Supplementary file 1 — supplementary information, Fig S1 [file 41422_2020_412_MOESM1_ESM.pdf]

Figure S1

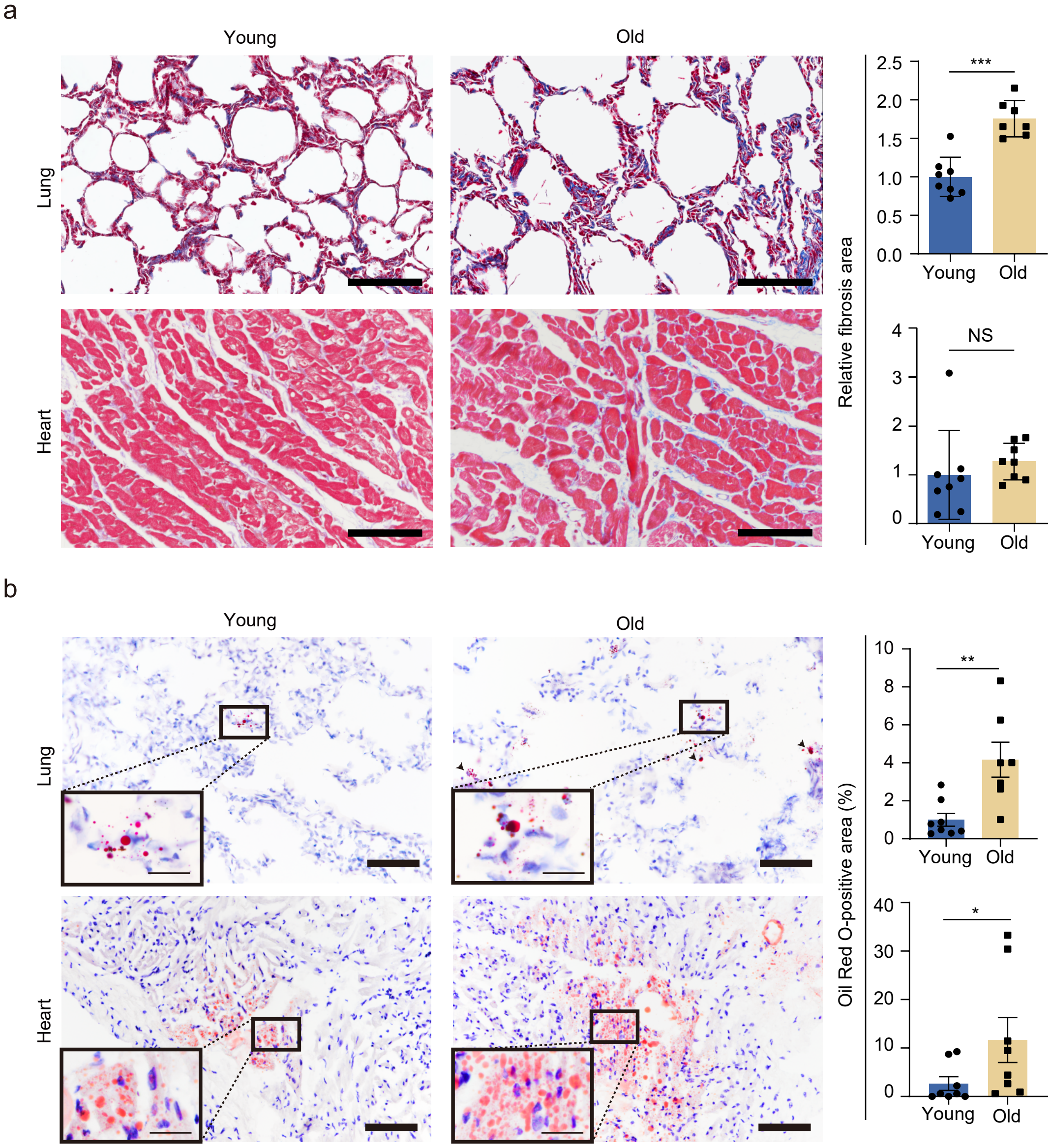
a
Young
Old
Lung
Heart
Relative fibrosis area
2.5
2.0
1.5
1.0
0.5
0.0
***
Young
Old
4
3
2
1
0
NS
Young
Old
b
Young
Old
Lung
Heart
Oil Red O-positive area (%)
10
8
6
4
2
0
**
Young
Old
40
30
20
10
0
*
Young
Old

Supplementary information, Figure S1. Morphological and histological characterizations of lung, heart, and aorta tissues from young and old monkeys.

a Masson-stained sections of lung and heart tissues from young and old monkeys. Representative images are shown on the left, and relative fibrosis area in the monkey lung and heart tissues are shown as the means ± SEM on the right. Scale bar, 100 μm. Young, $n = 8$ monkeys; old, $n = 7$ monkeys (lung). Young, $n = 8$; old, $n = 8$ monkeys (heart). *** $P < 0.001$. NS, not significant. **b** Oil Red O staining analysis of lung and heart tissues from young and old monkeys. Representative images are shown on the left, and Oil Red O-positive area for each tissue is shown as the means ± SEM to the right. The black rectangle represents Oil Red O staining positive areas. Scale bar, 100 μm and 10 μm (zoomed-in image). Young, $n = 8$; old, $n = 7$ monkeys (lung). Young, $n = 8$ monkeys; old, $n = 8$ monkeys (heart). ** $P < 0.01$, * $P < 0.05$.
