# Supplementary material for: Single-cell transcriptomic atlas of primate cardiopulmonary aging
Source: Cell Res. 2020 Sep 10;31(4):415–32. doi: 10.1038/s41422-020-00412-6 (PMC7483052; doi:10.1038/s41422-020-00412-6)
Supplement: Supplementary file 2 — supplementary information, Fig S2 [file 41422_2020_412_MOESM2_ESM.pdf]

Figure S2

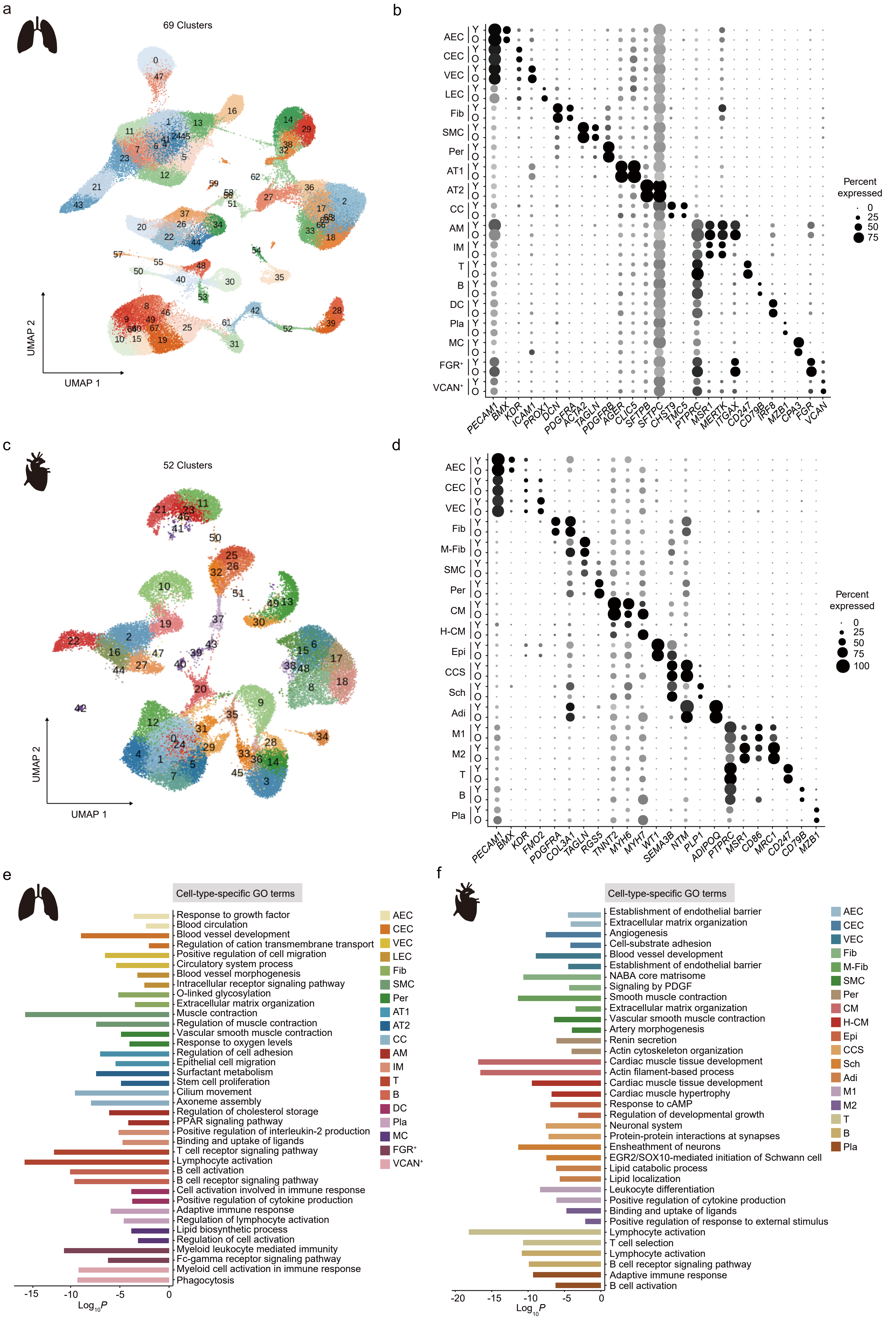

a
69 Clusters
UMAP 1
UMAP 2
b
AEC
CEC
VEC
LEC
Fib
SMC
Per
AT1
AT2
CC
AM
IM
T
B
DC
Pla
MC
FGR⁺
VCAN⁺
PECAM1 BMX KDR ICAM1 PROX1 DCN PDGFRA ACTA2 TAGLN PDGFRB AGER CLIC5 SFTPB SFTPC CHST9 TMC5 PTPRC MSR1 MERTK ITGAX CD247 CD79B IRF8 MZB1 CPA3 FGR VCAN
Percent expressed
0
25
50
75
c
52 Clusters
UMAP 1
UMAP 2
d
AEC
CEC
VEC
Fib
M-Fib
SMC
Per
CM
H-CM
Epi
CCS
Sch
Adi
M1
M2
T
B
Pla
PECAM1 BMX KDR FMO2 PDGFRA COL3A1 TAGLN RGS5 TNNT2 MYH6 MYH7 WT1 SEMA3B NTM PLP1 ADIPOQ PTPRC MSR1 CD86 MRC1 CD247 CD79B MZB1
Percent expressed
0
25
50
75
100
e
Cell-type-specific GO terms
Response to growth factor
Blood circulation
Blood vessel development
Regulation of cation transmembrane transport
Positive regulation of cell migration
Circulatory system process
Blood vessel morphogenesis
Intracellular receptor signaling pathway
O-linked glycosylation
Extracellular matrix organization
Muscle contraction
Regulation of muscle contraction
Vascular smooth muscle contraction
Response to oxygen levels
Regulation of cell adhesion
Epithelial cell migration
Surfactant metabolism
Stem cell proliferation
Cilium movement
Axoneme assembly
Regulation of cholesterol storage
PPAR signaling pathway
Positive regulation of interleukin-2 production
Binding and uptake of ligands
T cell receptor signaling pathway
Lymphocyte activation
B cell activation
B cell receptor signaling pathway
Cell activation involved in immune response
Positive regulation of cytokine production
Adaptive immune response
Regulation of lymphocyte activation
Lipid biosynthetic process
Regulation of cell activation
Myeloid leukocyte mediated immunity
Fc-gamma receptor signaling pathway
Myeloid cell activation in immune response
Phagocytosis
AEC
CEC
VEC
LEC
Fib
SMC
Per
AT1
AT2
CC
AM
IM
T
B
DC
Pla
MC
FGR⁺
VCAN⁺
-15 -10 -5 0
Log10P
f
Cell-type-specific GO terms
Establishment of endothelial barrier
Extracellular matrix organization
Angiogenesis
Cell-substrate adhesion
Blood vessel development
Establishment of endothelial barrier
NABA core matrisome
Signaling by PDGF
Smooth muscle contraction
Extracellular matrix organization
Vascular smooth muscle contraction
Artery morphogenesis
Renin secretion
Actin cytoskeleton organization
Cardiac muscle tissue development
Actin filament-based process
Cardiac muscle tissue development
Cardiac muscle hypertrophy
Response to cAMP
Regulation of developmental growth
Neuronal system
Protein-protein interactions at synapses
Ensheathment of neurons
EGR2/SOX10-mediated initiation of Schwann cell
Lipid catabolic process
Lipid localization
Leukocyte differentiation
Positive regulation of cytokine production
Binding and uptake of ligands
Positive regulation of response to external stimulus
Lymphocyte activation
T cell selection
Lymphocyte activation
B cell receptor signaling pathway
Adaptive immune response
B cell activation
AEC
CEC
VEC
Fib
M-Fib
SMC
Per
CM
H-CM
Epi
CCS
Sch
Adi
M1
M2
T
B
Pla
-20 -15 -10 -5 0
Log10P

Supplementary information, Figure S2. Characteristics of different cell types of monkey lung and heart.

a UMAP plot showing the cell clusters in monkey lung. 69 clusters were identified in 19 major cell types with classic cell-type-specific markers in the lung. **b** Dot plot showing the expression levels of specific marker genes for each cell type in young and old groups of monkey lung. **c** UMAP plot showing the cell clusters in monkey heart. 52 clusters were identified in 18 major cell types with classic cell-type-specific markers in the heart. **d** Dot plot showing the expression levels of specific marker genes for each cell type in young and old groups of monkey heart. **e** Bar plot showing the enriched GO terms (Biological Process) for top 30 marker genes of each cell type in lung highlighted by different colors corresponding to Fig. 1d. **f** Bar plot showing the enriched GO terms (Biological Process) for top 30 marker genes of each cell type in heart highlighted by different colors corresponding to Fig. 1g.
