# Supplementary material for: Single-cell transcriptomic atlas of primate cardiopulmonary aging
Source: Cell Res. 2020 Sep 10;31(4):415–32. doi: 10.1038/s41422-020-00412-6 (PMC7483052; doi:10.1038/s41422-020-00412-6)
Supplement: Supplementary file 3 — supplementary information, Fig S3 [file 41422_2020_412_MOESM3_ESM.pdf]

Figure S3

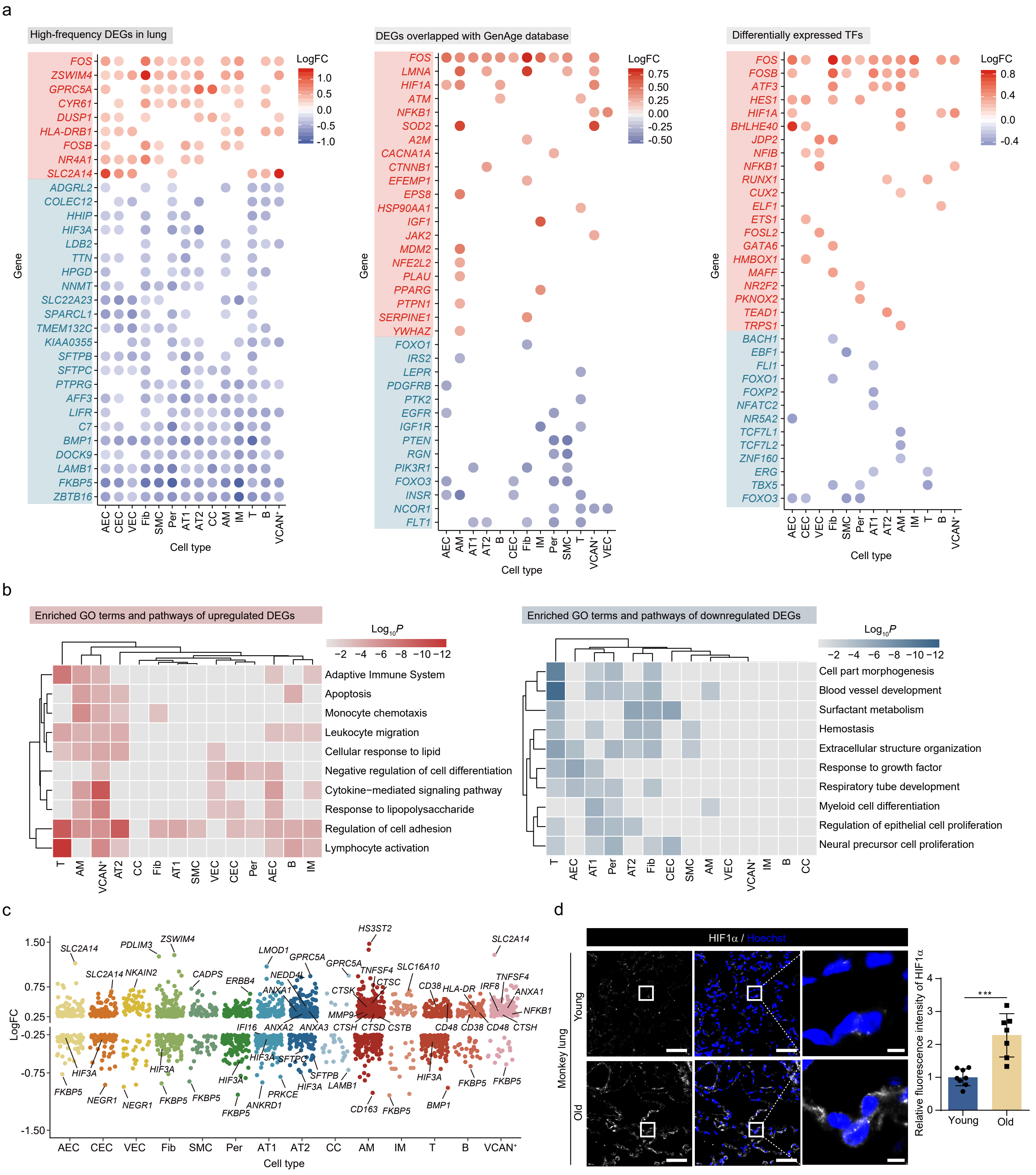

a
High-frequency DEGs in lung
LogFC
1.0
0.5
0.0
-0.5
-1.0
Gene
FOS
ZSWIM4
GPRC5A
CYR61
DUSP1
HLA-DRB1
FOSB
NR4A1
SLC2A14
ADGRL2
COLEC12
HHIP
HIF3A
LDB2
TTN
HPGD
NNMT
SLC22A23
SPARCL1
TMEM132C
KIAA0355
SFTPB
SFTPC
PTPRG
AFF3
LIFR
C7
BMP1
DOCK9
LAMB1
FKBP5
ZBTB16
AEC CEC VEC Fib SMC Per AT1 AT2 CC AM IM T B VCAN⁺
Cell type
DEGs overlapped with GenAge database
LogFC
0.75
0.50
0.25
0.00
-0.25
-0.50
FOS
LMNA
HIF1A
ATM
NFKB1
SOD2
A2M
CACNA1A
CTNNB1
EFEMP1
EPS8
HSP90AA1
IGF1
JAK2
MDM2
NFE2L2
PLAU
PPARG
PTPN1
SERPINE1
YWHAZ
FOXO1
IRS2
LEPR
PDGFRB
PTK2
EGFR
IGF1R
PTEN
RGN
PIK3R1
FOXO3
INSR
NCOR1
FLT1
AEC AM AT1 AT2 B CEC Fib IM Per SMC T VCAN⁺ VEC
Cell type
Differentially expressed TFs
LogFC
0.8
0.4
0.0
-0.4
FOS
FOSB
ATF3
HES1
HIF1A
BHLHE40
JDP2
NFIB
NFKB1
RUNX1
CUX2
ELF1
ETS1
FOSL2
GATA6
HMBOX1
MAFF
NR2F2
PKNOX2
TEAD1
TRPS1
BACH1
EBF1
FLI1
FOXO1
FOXP2
NFATC2
NR5A2
TCF7L1
TCF7L2
ZNF160
ERG
TBX5
FOXO3
AEC CEC VEC Fib SMC Per AT1 AT2 AM IM T B VCAN⁺
Cell type
b
Enriched GO terms and pathways of upregulated DEGs
Log10P
−2 −4 −6 −8 −10 −12
Adaptive Immune System
Apoptosis
Monocyte chemotaxis
Leukocyte migration
Cellular response to lipid
Negative regulation of cell differentiation
Cytokine−mediated signaling pathway
Response to lipopolysaccharide
Regulation of cell adhesion
Lymphocyte activation
T AM VCAN⁺ AT2 CC Fib AT1 SMC VEC CEC Per AEC B IM
Enriched GO terms and pathways of downregulated DEGs
Log10P
−2 −4 −6 −8 −10 −12
Cell part morphogenesis
Blood vessel development
Surfactant metabolism
Hemostasis
Extracellular structure organization
Response to growth factor
Respiratory tube development
Myeloid cell differentiation
Regulation of epithelial cell proliferation
Neural precursor cell proliferation
T AEC AT1 Per AT2 Fib CEC SMC AM VEC VCAN⁺ IM B CC
c
LogFC
1.50
0.75
0.25
-0.25
-0.75
-1.50
SLC2A14
PDLIM3
ZSWIM4
SLC2A14
NKAIN2
CADPS
ERBB4
LMOD1
GPRC5A
NEDD4L
ANXA1
GPRC5A
HS3ST2
TNFSF4
SLC16A10
CTSK
CTSC
MMP9
CD38
HLA-DR
IRF8
TNFSF4
ANXA1
NFKB1
SLC2A14
IFI16
ANXA2
ANXA3
CTSH
CTSD
CSTB
CD48
CD38
CD48
CTSH
HIF3A
HIF3A
HIF3A
HIF3A
SFTPC
SFTPB
HIF3A
LAMB1
HIF3A
FKBP5
NEGR1
NEGR1
FKBP5
FKBP5
FKBP5
ANKRD1
PRKCE
CD163
FKBP5
BMP1
FKBP5
FKBP5
AEC CEC VEC Fib SMC Per AT1 AT2 CC AM IM T B VCAN⁺
Cell type
d
HIF1α / Hoechst
Monkey lung
Young
Old
Relative fluorescence intensity of HIF1α
4
3
2
1
0
***
Young
Old

Supplementary information, Figure S3. Age-related transcriptional alterations in various cell types of monkey lung.

a Left, dot plot showing the DEGs with high-frequency across various cell types in monkey lung. Middle, dot plot showing the DEGs overlapping with the GenAge database (https://genomics.senescence.info/genes/) in monkey lung. Right, dot plot showing differentially expressed TFs across different cell types in monkey lung. Red denotes upregulated genes; blue denotes downregulated genes. **b** Heatmaps showing the enriched GO terms (Biological Process) or pathways of upregulated and downregulated DEGs across different cell types in monkey lung. **c** Scatter plot showing the aging-related DEGs across 14 lung cell types ($|\log FC| > 0.25$, adjusted P value < 0.05). **d** Immunofluorescence staining of HIF1α in lung tissues from young and old monkeys. Quantitative data are shown as the means ± SEM. Young, $n = 8$ monkeys; old, $n = 7$ monkeys. Scale bar, 50 μm and 5 μm (zoomed-in image). *** $P < 0.001$.
