# Supplementary material for: Single-cell transcriptomic atlas of primate cardiopulmonary aging
Source: Cell Res. 2020 Sep 10;31(4):415–32. doi: 10.1038/s41422-020-00412-6 (PMC7483052; doi:10.1038/s41422-020-00412-6)
Supplement: Supplementary file 4 — supplementary information, Fig S4 [file 41422_2020_412_MOESM4_ESM.pdf]

Figure S4

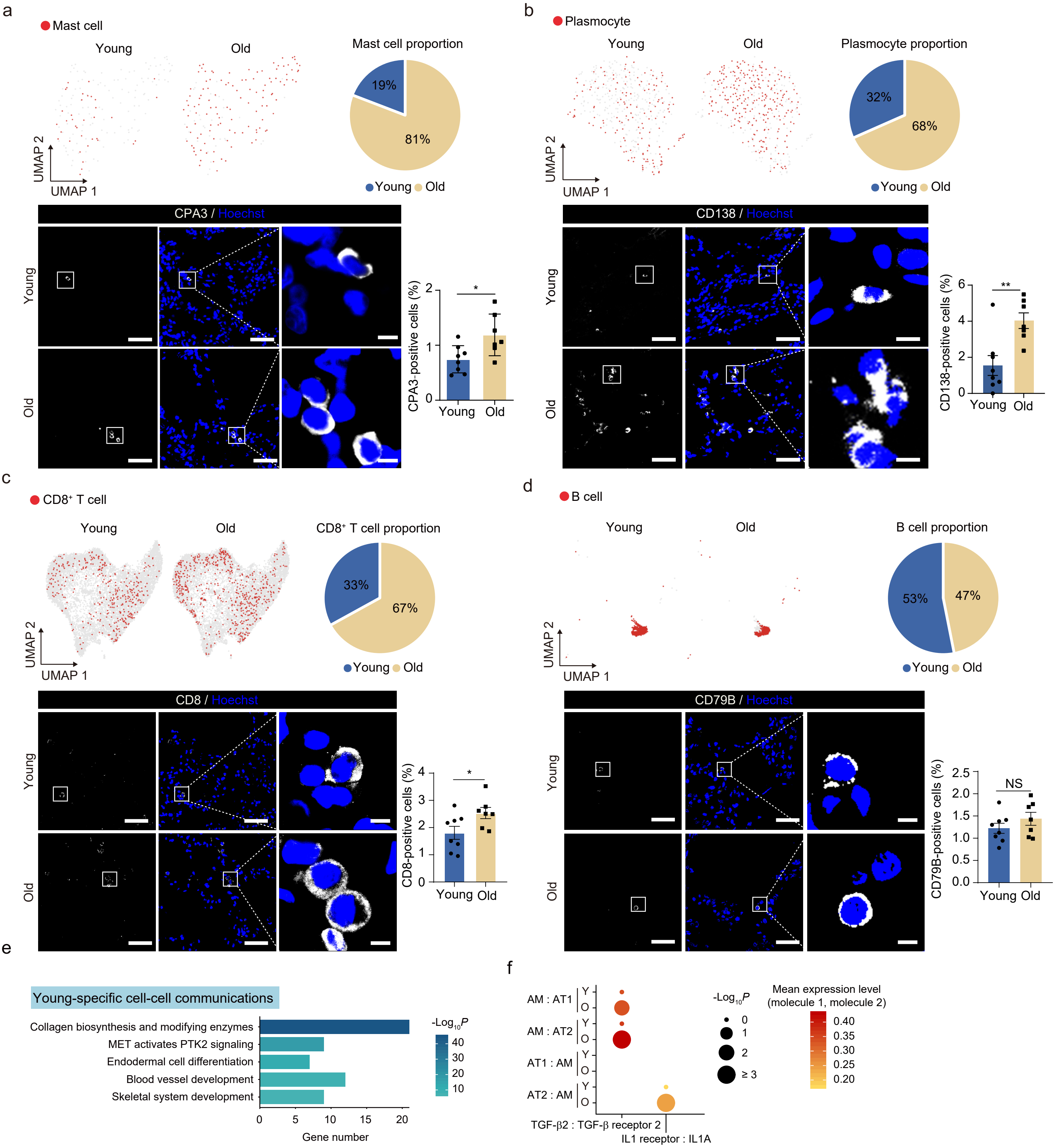
a
Mast cell
Young
Old
Mast cell proportion
19%
81%
UMAP 2
UMAP 1
Young Old
CPA3 / Hoechst
Young
Old
CPA3-positive cells (%)
*
b
Plasmocyte
Young
Old
Plasmocyte proportion
32%
68%
CD138 / Hoechst
CD138-positive cells (%)
**
c
CD8+ T cell
CD8+ T cell proportion
33%
67%
CD8 / Hoechst
CD8-positive cells (%)
d
B cell
B cell proportion
53%
47%
CD79B / Hoechst
CD79B-positive cells (%)
NS
e
Young-specific cell-cell communications
Collagen biosynthesis and modifying enzymes
MET activates PTK2 signaling
Endodermal cell differentiation
Blood vessel development
Skeletal system development
Gene number
-Log10P
f
AM : AT1
AM : AT2
AT1 : AM
AT2 : AM
Y
O
TGF-β2 : TGF-β receptor 2
IL1 receptor : IL1A
-Log10P
Mean expression level (molecule 1, molecule 2)

Supplementary information, Figure. S4. Immunofluorescence staining of different immune cell types in monkey lung.

a Top left, UMAP plots showing mast cells in young and old groups of monkey lung. Top right, pie plot showing proportions of mast cells in young and old groups of monkey lung. Bottom, immunofluorescence staining of the mast cell marker CPA3 in lung tissues from young and old monkeys. Representative images are shown on the left, and quantitative data are shown as the means ± SEM on the right. Scale bar, 50 μm and 5 μm. Young, $n = 8$ monkeys; old, $n = 7$ monkeys. * $P < 0.05$. **b** Top left, UMAP plots showing the plasmocytes in young and old groups of monkey lung. Top right, pie plot showing the proportions of plasmocytes in young and old groups of monkey lung. Bottom, immunofluorescence staining of the plasmocyte marker CD138 in lung tissues from young and old monkeys. Representative images are shown on the left, and quantitative data are shown as the means ± SEM on the right. Scale bar, 50 μm and 5 μm. Young, $n = 8$ monkeys; old, $n = 7$ monkeys. * $P < 0.05$. **c** Top left, UMAP plots showing $CD8^+$ T cells in young and old groups of monkey lung. Top right, pie plot showing proportions of $CD8^+$ T cells in young and old groups of monkey lung. Bottom, immunofluorescence staining of $CD8^+$ cells in lung tissues from young and old monkeys. Representative images are shown on the left, and quantitative data are shown as the means ± SEM on the right. Scale bar, 50 μm and 5 μm. Young, $n = 8$ monkeys; old, $n = 7$ monkeys. ** $P < 0.01$. **d** Top left, UMAP plots showing the B cells in young and old groups of monkey lung. Top right, pie plot showing the proportions of B cells in young and old groups of monkey lung. Bottom, immunofluorescence staining of the B cell marker CD79B in lung tissues from young and old monkeys showing no change in the number of B cells with age, consistent with the snRNA-seq results. Representative images are shown on the left, and quantitative data are shown as the means ± SEM on the right. Scale bar, 50 μm and 5 μm. Young, $n = 8$ monkeys; old, $n = 7$ monkeys. NS, not significant. **e** Bar plot showing the functional enrichment analysis of young-specific cell-cell communications in monkey lung. **f** Dot plot showing the old-specific ligand-receptor interactions between AM and alveolar epithelial cells in
