# Supplementary material for: Single-cell transcriptomic atlas of primate cardiopulmonary aging
Source: Cell Res. 2020 Sep 10;31(4):415–32. doi: 10.1038/s41422-020-00412-6 (PMC7483052; doi:10.1038/s41422-020-00412-6)
Supplement: Supplementary file 5 — supplementary information, Fig S5 [file 41422_2020_412_MOESM5_ESM.pdf]

Figure S5

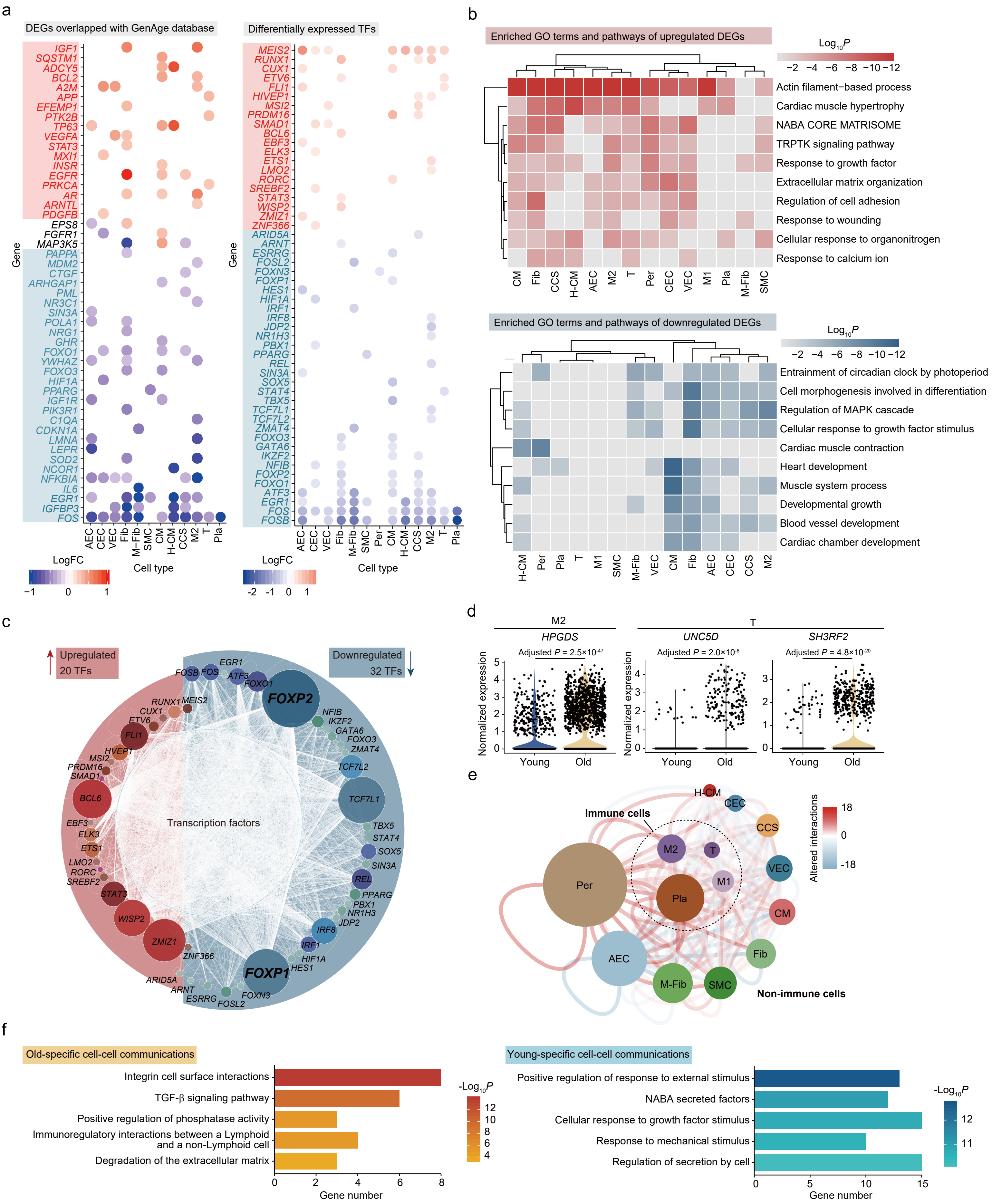
a
DEGs overlapped with GenAge database
Differentially expressed TFs
Gene
IGF1 SQSTM1 ADCY5 BCL2 A2M APP EFEMP1 PTK2B TP63 VEGFA STAT3 MXI1 INSR EGFR PRKCA AR ARNTL PDGFB EPS8 FGFR1 MAP3K5 PAPPA MDM2 CTGF ARHGAP1 PML NR3C1 SIN3A POLA1 NRG1 GHR FOXO1 YWHAZ FOXO3 HIF1A PPARG IGF1R PIK3R1 C1QA CDKN1A LMNA LEPR SOD2 NCOR1 NFKBIA IL6 EGR1 IGFBP3 FOS
MEIS2 RUNX1 CUX1 ETV6 FLI1 HIVEP1 MSI2 PRDM16 SMAD1 BCL6 EBF3 ELK3 ETS1 LMO2 RORC SREBF2 STAT3 WISP2 ZMIZ1 ZNF366 ARID5A ARNT ESRRG FOSL2 FOXN3 FOXP1 HES1 HIF1A IRF1 IRF8 JDP2 NR1H3 PBX1 PPARG REL SIN3A SOX5 STAT4 TBX5 TCF7L1 TCF7L2 ZMAT4 FOXO3 GATA6 IKZF2 NFIB FOXP2 FOXO1 ATF3 EGR1 FOS FOSB
AEC CEC VEC Fib M-Fib SMC CM H-CM CCS M2 T Pla
AEC CEC VEC Fib M-Fib SMC Per CM H-CM CCS M2 T Pla
LogFC
Cell type
−1 0 1
-2 -1 0 1
b
Enriched GO terms and pathways of upregulated DEGs
Log10P
−2 −4 −6 −8 −10 −12
Actin filament−based process
Cardiac muscle hypertrophy
NABA CORE MATRISOME
TRPTK signaling pathway
Response to growth factor
Extracellular matrix organization
Regulation of cell adhesion
Response to wounding
Cellular response to organonitrogen
Response to calcium ion
CM Fib CCS H-CM AEC M2 T Per CEC VEC M1 Pla M-Fib SMC
Enriched GO terms and pathways of downregulated DEGs
Log10P
−2 −4 −6 −8 −10 −12
Entrainment of circadian clock by photoperiod
Cell morphogenesis involved in differentiation
Regulation of MAPK cascade
Cellular response to growth factor stimulus
Cardiac muscle contraction
Heart development
Muscle system process
Developmental growth
Blood vessel development
Cardiac chamber development
H-CM Per Pla T M1 SMC M-Fib VEC CM Fib AEC CEC CCS M2
c
Upregulated
20 TFs
Downregulated
32 TFs
Transcription factors
FOXP2 FOXP1 TCF7L1 BCL6 ZMIZ1 WISP2 STAT3 FLI1
d
M2
T
HPGDS
UNC5D
SH3RF2
Adjusted P = 2.5×10^-47
Adjusted P = 2.0×10^-8
Adjusted P = 4.8×10^-20
Normalized expression
Young Old
e
Immune cells
Non-immune cells
H-CM CEC CCS VEC CM Fib SMC M-Fib AEC Per Pla M2 T M1
Altered interactions
18 0 -18
f
Old-specific cell-cell communications
Integrin cell surface interactions
TGF-β signaling pathway
Positive regulation of phosphatase activity
Immunoregulatory interactions between a Lymphoid and a non-Lymphoid cell
Degradation of the extracellular matrix
Gene number
0 2 4 6 8
-Log10P
12 10 8 6 4
Young-specific cell-cell communications
Positive regulation of response to external stimulus
NABA secreted factors
Cellular response to growth factor stimulus
Response to mechanical stimulus
Regulation of secretion by cell
Gene number
0 5 10 15
-Log10P
12 11

Supplementary information, Figure S5. Age-related transcriptional alterations in various cell types of monkey heart.

a Left, dot plot showing the DEGs overlapped with the GenAge database (https://genomics.senescence.info/genes/) in monkey heart. Right, dot plot showing differentially expressed TFs across different cell types in monkey heart. Red denotes upregulated genes; blue denotes downregulated genes. **b** Heatmaps showing the enriched GO terms (Biological Process) or pathways of upregulated (top) and downregulated (bottom) DEGs across different cell types in monkey heart. **c** Network plot showing differentially expressed TFs during aging (|logFC| > 0.25, adjusted *P* value < 0.05). The node size is positively correlated with the number of target genes. Red nodes represent upregulated TFs, blue nodes represent downregulated TFs. **d** Violin plots showing the expression levels of *HPGDS, UNC5D,* and *SH3RF2* in M2 and T cells of monkey heart across young and old groups. **e** Network plot showing the cell-cell communications between immune cells and non-immune cells in monkey heart The color of connecting lines indicates the number of altered interaction pairs. Red, increased interactions; blue, decreased interactions. **f** Bar plot showing the functional enrichment of old-specific (left) and young-specific (right) cell-cell communications.
