# Supplementary material for: Single-cell transcriptomic atlas of primate cardiopulmonary aging
Source: Cell Res. 2020 Sep 10;31(4):415–32. doi: 10.1038/s41422-020-00412-6 (PMC7483052; doi:10.1038/s41422-020-00412-6)
Supplement: Supplementary file 6 — supplementary information, Fig S6 [file 41422_2020_412_MOESM6_ESM.pdf]

Figure S6

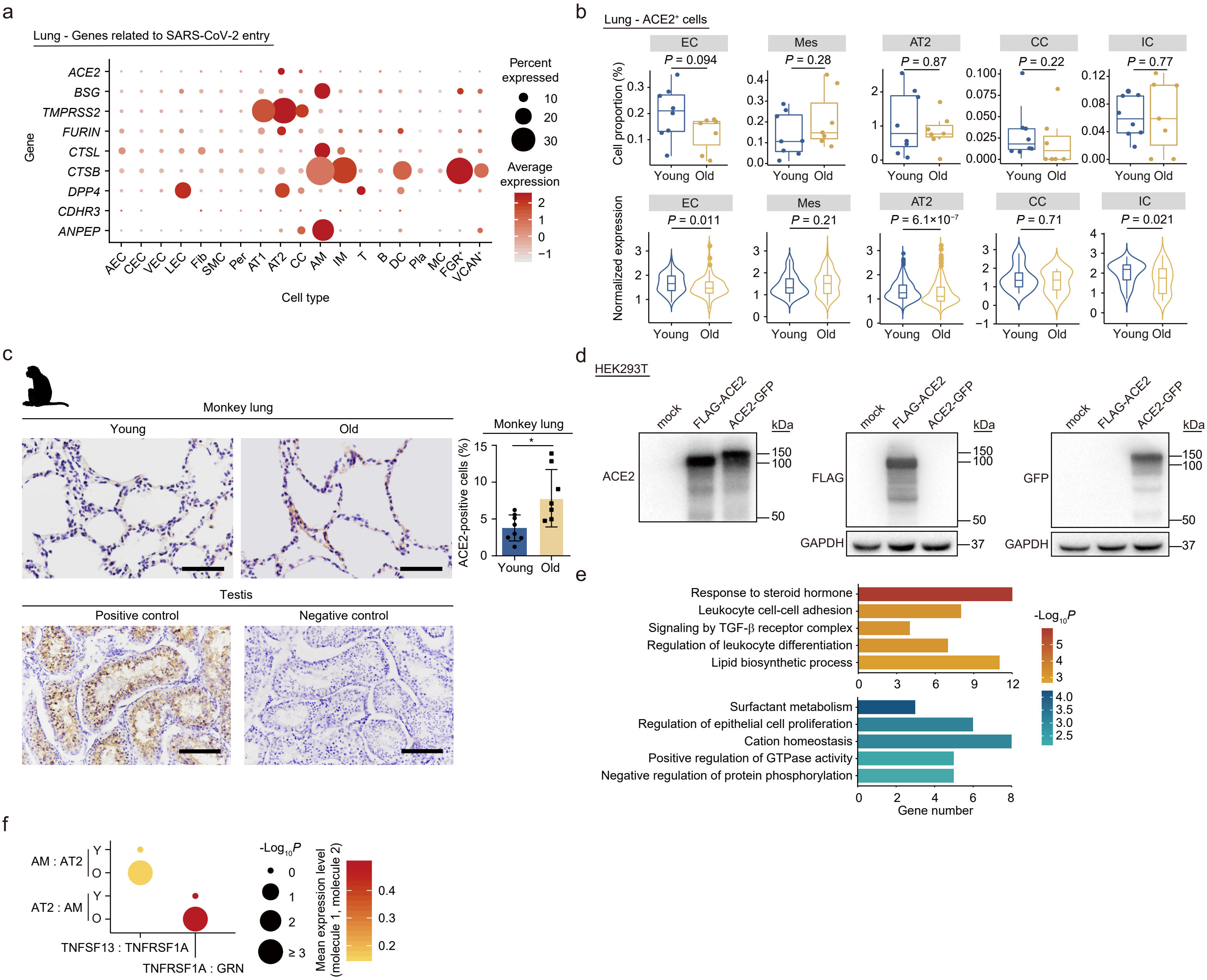
a
Lung - Genes related to SARS-CoV-2 entry
Gene
ACE2
BSG
TMPRSS2
FURIN
CTSL
CTSB
DPP4
CDHR3
ANPEP
AEC CEC VEC LEC Fib SMC Per AT1 AT2 CC AM IM T B DC Pla MC FGR⁺ VCAN⁺
Cell type
Percent expressed
10
20
30
Average expression
2
1
0
−1
b
Lung - ACE2⁺ cells
EC
Mes
AT2
CC
IC
P = 0.094
P = 0.28
P = 0.87
P = 0.22
P = 0.77
Cell proportion (%)
Young Old
P = 0.011
P = 0.21
P = 6.1×10⁻⁷
P = 0.71
P = 0.021
Normalized expression
c
Monkey lung
Young
Old
Monkey lung
ACE2-positive cells (%)
*
Testis
Positive control
Negative control
d
HEK293T
mock
FLAG-ACE2
ACE2-GFP
kDa
ACE2
FLAG
GFP
150
100
50
37
GAPDH
e
Response to steroid hormone
Leukocyte cell-cell adhesion
Signaling by TGF-β receptor complex
Regulation of leukocyte differentiation
Lipid biosynthetic process
Surfactant metabolism
Regulation of epithelial cell proliferation
Cation homeostasis
Positive regulation of GTPase activity
Negative regulation of protein phosphorylation
Gene number
-Log10P
f
AM : AT2
AT2 : AM
Y
O
TNFSF13 : TNFRSF1A
TNFRSF1A : GRN
-Log10P
0
1
2
≥ 3
Mean expression level (molecule 1, molecule 2)
0.4
0.3
0.2

Supplementary information, Figure S6. Age-related transcriptional alterations in SARS-CoV-2 target cell types of monkey lung.

a Dot plot showing expression levels of genes associated with SARS-CoV-2 entry across cell types in monkey lung. See legend of Fig. 1d for cell type abbreviations. **b** Box and violin plots showing the proportions of $ACE2^+$ cells and *ACE2* expression levels in indicated cell types of young and old monkey lungs. See the legend of Fig. 1d for cell type abbreviation. **c** Immunohistochemistry staining of ACE2 in lung tissues from young and old monkeys (left). Immunohistochemistry staining of IgG and ACE2 in monkey testis was used as negative and positive controls (bottom). Quantitative data to the right are shown as the means ± SEM. Scale bar, 50 μm (lung) and 100 μm (small intestine and testis). Young, n = 8 monkeys; old, n = 7 monkeys. * $P < 0.05$. **d** Expression of ACE2 (left), flag (middle) and GFP (right) in HEK293T cell transfected with FLAG-ACE2 and ACE2-GFP by western blotting. The specificity of the protein band confirmed the reliability of the anti-ACE2 antibody used for immunoblotting. **e** Bar plot showing the enriched GO terms (Biological Process) or pathway of DEGs ($|logFC| > 0.25$, adjusted P value < 0.05) in aged $ACE2^+$ cells. **f** Dot plot showing the old-specific ligand-receptor interactions between AM and $ACE2^+$ AT2 in monkey lung.
