# Supplementary material for: Single-cell transcriptomic atlas of primate cardiopulmonary aging
Source: Cell Res. 2020 Sep 10;31(4):415–32. doi: 10.1038/s41422-020-00412-6 (PMC7483052; doi:10.1038/s41422-020-00412-6)
Supplement: Supplementary file 7 — supplementary information, Fig S7 [file 41422_2020_412_MOESM7_ESM.pdf]

Figure S7

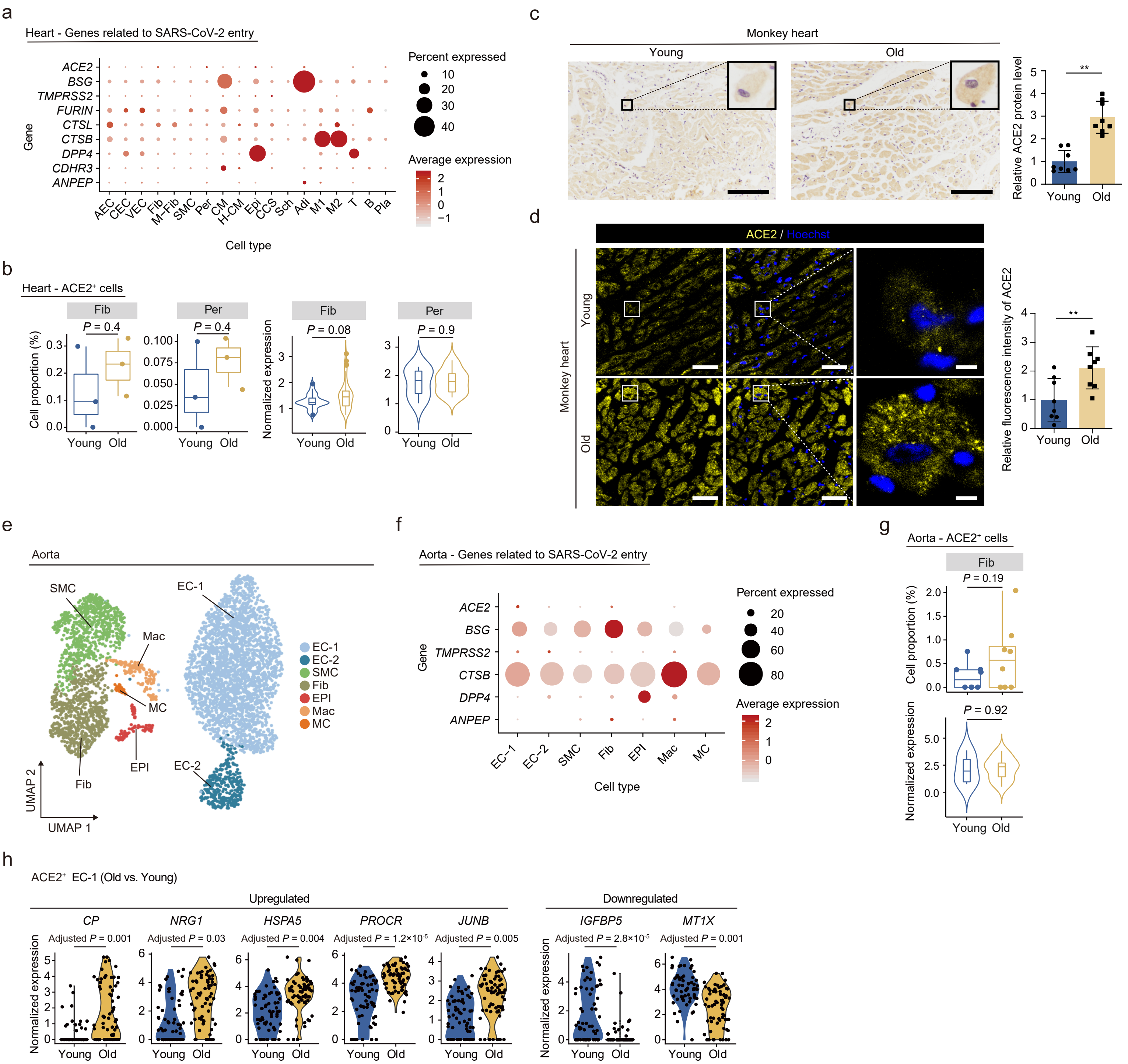

a
Heart - Genes related to SARS-CoV-2 entry
ACE2
BSG
TMPRSS2
FURIN
CTSL
CTSB
DPP4
CDHR3
ANPEP
Gene
AEC CEC VEC Fib M-Fib SMC Per CM H-CM Epi CCS Sch Adi M1 M2 T B Pla
Cell type
Percent expressed
10
20
30
40
Average expression
2
1
0
−1
b
Heart - ACE2⁺ cells
Fib
Per
P = 0.4
P = 0.4
P = 0.08
P = 0.9
Cell proportion (%)
Normalized expression
Young Old
c
Monkey heart
Young
Old
Relative ACE2 protein level
**
d
ACE2 / Hoechst
Monkey heart
Young
Old
Relative fluorescence intensity of ACE2
**
e
Aorta
SMC
EC-1
Mac
MC
EPI
Fib
EC-2
EC-1
EC-2
SMC
Fib
EPI
Mac
MC
UMAP 2
UMAP 1
f
Aorta - Genes related to SARS-CoV-2 entry
ACE2
BSG
TMPRSS2
CTSB
DPP4
ANPEP
Gene
EC−1 EC−2 SMC Fib EPI Mac MC
Cell type
Percent expressed
20
40
60
80
Average expression
2
1
0
g
Aorta - ACE2⁺ cells
Fib
P = 0.19
Cell proportion (%)
P = 0.92
Normalized expression
Young Old
h
ACE2⁺ EC-1 (Old vs. Young)
Upregulated
Downregulated
CP
NRG1
HSPA5
PROCR
JUNB
IGFBP5
MT1X
Adjusted P = 0.001
Adjusted P = 0.03
Adjusted P = 0.004
Adjusted P = 1.2×10⁻⁵
Adjusted P = 0.005
Adjusted P = 2.8×10⁻⁵
Adjusted P = 0.001
Normalized expression
Young Old

Supplementary information, Figure S7. Age-related transcriptional alterations in SARS-CoV-2 target cell types of monkey cardiovascular system.

a Dot plot showing expression levels of genes associated with SARS-CoV-2 entry across cell types in monkey hearts. See the legend of Fig. 1g for cell type abbreviations. **b** Box and violin plots showing the proportions of $ACE2^{+}$ cells and *ACE2* expression levels in Fib and Per of young and old monkey hearts. See the legend of Fig. 1g for cell type abbreviations. **c** Immunohistochemistry staining of ACE2 in heart tissues from young and old monkeys (left). Quantitative data to the right are shown as the means ± SEM. Scale bar, 100 µm. Young, $n = 8$ monkeys; old, $n = 8$ monkeys. ** $P < 0.01$. **d** Immunofluorescence staining of ACE2 in heart tissues from young and old monkeys (left). Quantitative data to the right are shown as the means ± SEM. Scale bar, 50 µm and 5 µm (zoomed-in image). Young, $n = 8$ monkeys; old, $n = 8$ monkeys. ** $P < 0.01$. **e** UMAP plot showing the different cell types in cynomolgus monkey aorta. EC-1, endothelial cells-1; EC-2, endothelial cells-2; SMC, smooth muscle cells; Fib, fibroblasts; EPI, epithelial cells; Mac, macrophages; MC, mast cells. **f** Dot plot showing expression levels of genes associated with SARS-CoV-2 entry across cell types in monkey aorta. See the legend of Supplementary information, Fig. S7e for cell type abbreviations. **g** Box and violin plots showing the proportions of $ACE2^{+}$ cells and *ACE2* expression levels in Fib of young and old monkey aortas. **h** Violin plots showing expression levels of upregulated and downregulated DEGs in $ACE2^{+}$ EC-1 during aging in monkey aorta.
