# Supplementary material for: Single-cell transcriptomic atlas of primate cardiopulmonary aging
Source: Cell Res. 2020 Sep 10;31(4):415–32. doi: 10.1038/s41422-020-00412-6 (PMC7483052; doi:10.1038/s41422-020-00412-6)
Supplement: Supplementary file 8 — supplementary information, Fig S8 [file 41422_2020_412_MOESM8_ESM.pdf]

Figure S8

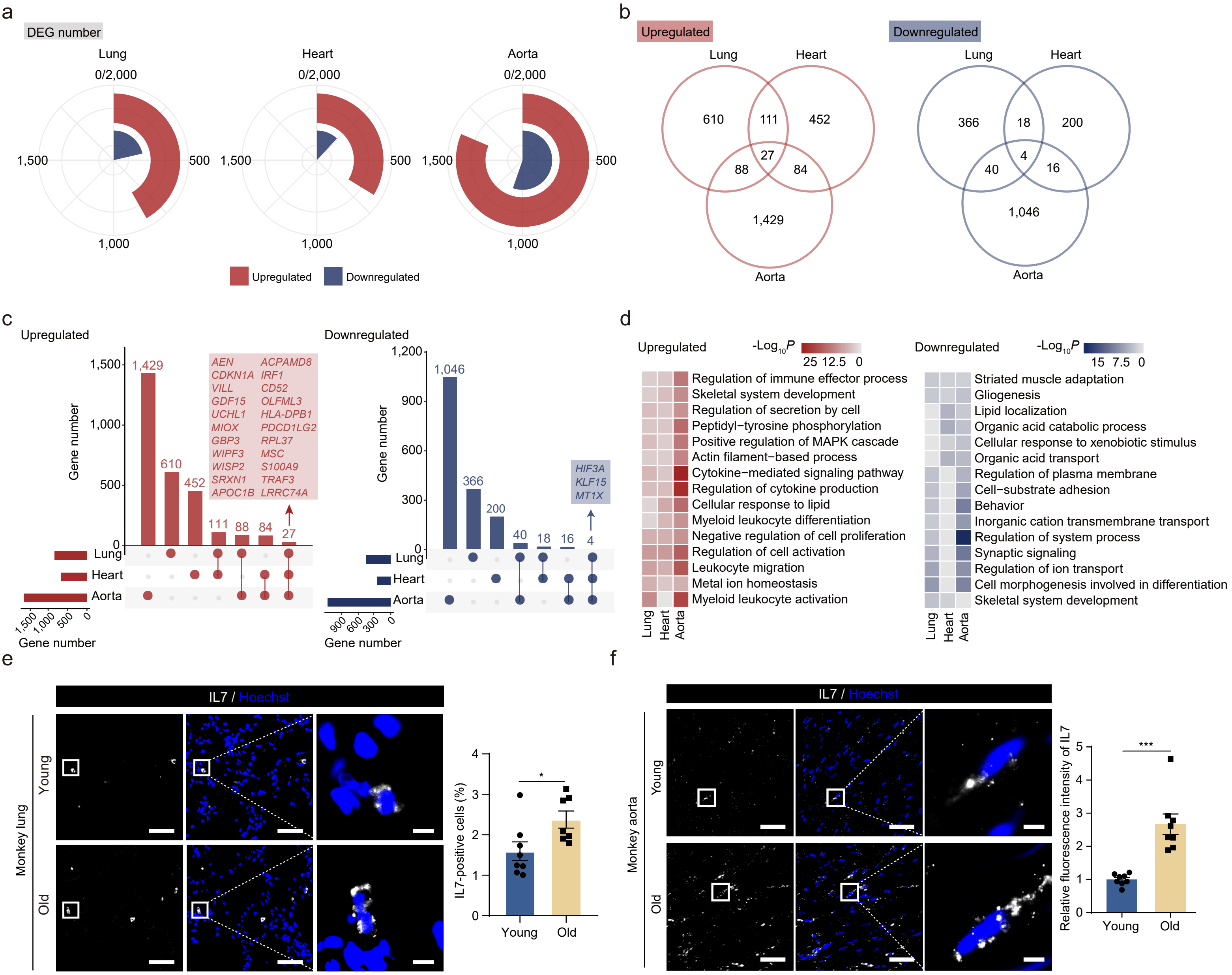
a
DEG number
Lung
Heart
Aorta
0/2,000
1,500
500
1,000
Upregulated
Downregulated
b
Upregulated
Downregulated
610
111
452
27
88
84
1,429
366
18
200
4
40
16
1,046
c
Upregulated
Downregulated
Gene number
1,429
610
452
111
88
84
27
AEN
ACPAMD8
CDKN1A
IRF1
VILL
CD52
GDF15
OLFML3
UCHL1
HLA-DPB1
MIOX
PDCD1LG2
GBP3
RPL37
WIPF3
MSC
WISP2
S100A9
SRXN1
TRAF3
APOC1B
LRRC74A
1,046
366
200
40
18
16
4
HIF3A
KLF15
MT1X
d
Upregulated
Downregulated
-Log10P
25 12.5 0
15 7.5 0
Regulation of immune effector process
Skeletal system development
Regulation of secretion by cell
Peptidyl−tyrosine phosphorylation
Positive regulation of MAPK cascade
Actin filament−based process
Cytokine−mediated signaling pathway
Regulation of cytokine production
Cellular response to lipid
Myeloid leukocyte differentiation
Negative regulation of cell proliferation
Regulation of cell activation
Leukocyte migration
Metal ion homeostasis
Myeloid leukocyte activation
Striated muscle adaptation
Gliogenesis
Lipid localization
Organic acid catabolic process
Cellular response to xenobiotic stimulus
Organic acid transport
Regulation of plasma membrane
Cell−substrate adhesion
Behavior
Inorganic cation transmembrane transport
Regulation of system process
Synaptic signaling
Regulation of ion transport
Cell morphogenesis involved in differentiation
Skeletal system development
e
IL7 / Hoechst
Monkey lung
Young
Old
IL7-positive cells (%)
*
f
IL7 / Hoechst
Monkey aorta
Young
Old
Relative fluorescence intensity of IL7
***

Supplementary information, Figure S8. Bulk RNA-seq analysis of monkey lung, heart, and aorta.

a Pie plots showing the numbers of upregulated (red) and downregulated (blue) DEGs across different tissues in bulk RNA-seq data. **b** Venn plots showing the overlapping DEGs across different tissues in bulk RNA-seq data. Left, upregulated; right, downregulated. **c** Bar and dot plots showing the numbers of unique and overlapping DEGs across different tissues in bulk RNA-seq data. Left, upregulated; right, downregulated. **d** Heatmaps showing the functional enrichment of upregulated and downregulated DEGs across different tissues in bulk RNA-seq data. **e** Immunofluorescence staining of IL7 in lung tissues from young and old monkeys. Quantitative data are shown as the means ± SEM. Young, n = 8 monkeys; old, n = 7 monkeys. Scale bar, 50 μm and 5 μm (zoomed-in image). * $P < 0.05$. **f** Immunofluorescence staining of IL7 in aorta tissues from young and old monkeys. Quantitative data are shown as the means ± SEM. Young, n = 8 monkeys; old, n = 8 monkeys. Scale bar, 50 μm and 5 μm (zoomed-in image). *** $P < 0.001$.
