# Supplementary material for: Single-cell transcriptomic atlas of primate cardiopulmonary aging
Source: Cell Res. 2020 Sep 10;31(4):415–32. doi: 10.1038/s41422-020-00412-6 (PMC7483052; doi:10.1038/s41422-020-00412-6)
Supplement: Supplementary file 9 — supplementary information, Fig S9 [file 41422_2020_412_MOESM9_ESM.pdf]

Figure S9

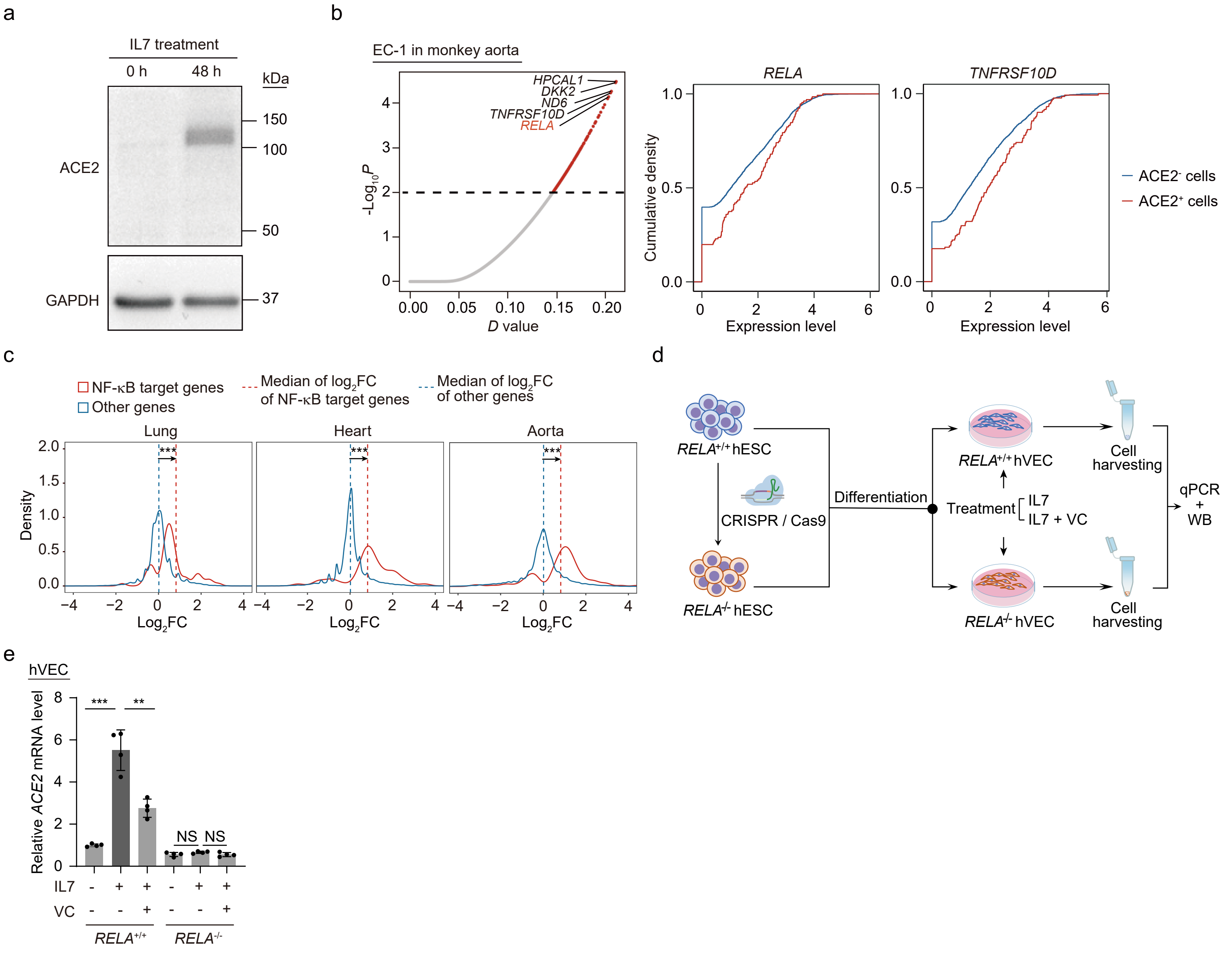

a
IL7 treatment
0 h
48 h
kDa
ACE2
150
100
50
GAPDH
37
b
EC-1 in monkey aorta
HPCAL1
DKK2
ND6
TNFRSF10D
RELA
-Log10P
D value
RELA
TNFRSF10D
Cumulative density
Expression level
ACE2- cells
ACE2+ cells
c
NF-κB target genes
Other genes
Median of log2FC of NF-κB target genes
Median of log2FC of other genes
Lung
Heart
Aorta
Density
Log2FC
d
RELA+/+ hESC
CRISPR / Cas9
RELA-/- hESC
Differentiation
RELA+/+ hVEC
RELA-/- hVEC
Treatment
IL7
IL7 + VC
Cell harvesting
qPCR + WB
e
hVEC
Relative ACE2 mRNA level
NS
IL7
VC
RELA+/+
RELA-/-

Supplementary information, Figure S9. A key role of RelA in mediating IL7-induced ACE2 expression.

a Western blot analysis of ACE2 expression after a 48-hr treatment with IL7 (10 ng/mL in HAECs. GAPDH was used as a loading control. **b** Left, the distribution of the differences in gene expression levels between *ACE2*-positive cells and *ACE2*-negative cells in EC-1 cells of aorta. Only five genes with the highest $-\log_{10}P$ values are labeled. *D* and *P* values were calculated with K-S test. Red points indicate genes with *P* values < 0.01. Right, cumulative distributions of the expression levels of *RELA* and *TNFRSF10D* in *ACE2*-positive cells (red) and *ACE2*-negative cells. **c** Density plot showing Log_2FC of NF-κB target genes between old and young tissues from bulk RNA-seq data of monkeys. *** $P < 0.001$ (two-sided Wilcoxon rank-sum test). **d** Schematic diagram for the generation of $RELA^{-/-}$ human embryonic stem cell and human vascular endothelial cell (hVEC) derivatives, followed by the treatment of $RELA^{+/+}$ and $RELA^{-/-}$ hVEC with IL7 and the subsequent detection of *ACE2* expression by RT-qPCR. **e** RT-qPCR showing the expression of *ACE2* after a 48-hr treatment with IL7 (10 ng/mL) and Vitamin C (280 μm) in $RELA^{+/+}$ and $RELA^{-/-}$ hVEC (passage 2). The data are shown as means ± SEM, $n = 4$. ** $P < 0.01$. *** $P < 0.001$. NS, not significant.
